# Modulation of the antagonistic properties of an insulin mimetic peptide by disulfide bridge modifications

**DOI:** 10.1002/psc.3478

**Published:** 2023-01-25

**Authors:** Marta Lubos, Jan Pícha, Irena Selicharová, Jíří Žák, Miloš Buděšínský, Katarína Mitrová, Lenka Žáková, Jiří Jiráček

**Affiliations:** ^1^ Institute of Organic Chemistry and Biochemistry Czech Academy of Sciences Praha Czech Republic

**Keywords:** antagonism, dicarba, disulfide mimetics, insulin mimetic peptide, insulin receptor, staple, triazole

## Abstract

Insulin is a peptide responsible for regulating the metabolic homeostasis of the organism; it elicits its effects through binding to the transmembrane insulin receptor (IR). Insulin mimetics with agonistic or antagonistic effects toward the receptor are an exciting field of research and could find applications in treating diabetes or malignant diseases. We prepared five variants of a previously reported 20‐amino acid insulin‐mimicking peptide. These peptides differ from each other by the structure of the covalent bridge connecting positions 11 and 18. In addition to the peptide with a disulfide bridge, a derivative with a dicarba bridge and three derivatives with a 1,2,3‐triazole differing from each other by the presence of sulfur or oxygen in their staples were prepared. The strongest binding to IR was exhibited by the peptide with a disulfide bridge. All other derivatives only weakly bound to IR, and a relationship between increasing bridge length and lower binding affinity can be inferred. Despite their nanomolar affinities, none of the prepared peptide mimetics was able to activate the insulin receptor even at high concentrations, but all mimetics were able to inhibit insulin‐induced receptor activation. However, the receptor remained approximately 30% active even at the highest concentration of the agents; thus, the agents behave as partial antagonists. An interesting observation is that these mimetic peptides do not antagonize insulin action in proportion to their binding affinities. The compounds characterized in this study show that it is possible to modulate the functional properties of insulin receptor peptide ligands using disulfide mimetics.

## Graphical abstract

Insulin mimetics with agonistic or antagonistic effects toward the receptor could find applications in treating diabetes or malignant diseases. As demonstrated, it is possible to modulate the functional properties of insulin receptor unnatural peptide ligands using disulfide bridge mimetics.

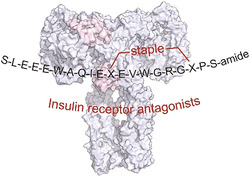

## 1 INTRODUCTION

Insulin binding to the insulin receptor (IR) and receptor activation are complex processes that have been the subject of intense mutagenesis and structural studies for decades.^1^ IR can be described as an (αβ)_2_ dimer consisting of two αβ domains, where the α subunits are extracellular and the β subunits are partially extracellular, membrane‐spanning, and intracellular. The dimeric and symmetric nature of IR is the reason for the existence of two binding regions for insulin, which interact with each other on the principle of negative cooperativity.^2^ The current view of insulin–receptor interactions assumes the existence of two insulin binding sites, Site 1 and Site 2, in each receptor binding region.^3^, ^4^ The coordinated interaction of insulin with both binding sites is essential for effective binding to the receptor and especially for its full activation.^5^, ^6^

Insulin is a hormone responsible for regulating the overall energy balance of the body and primarily for allowing the entry of glucose from the blood into the cells of muscle and adipose tissue.^7^ Insulin is a lifesaver for millions of diabetic patients worldwide.^8^ Insulin is a small protein and therefore cannot be given orally, and subcutaneous administration is the main way to give insulin to patients.^9^ Subcutaneous administration of insulin is accompanied by dosing and timing problems.^10^ The storage and distribution of pharmaceutical preparations of insulin in solution are also not without problems due to the tendency of insulin to aggregate at higher temperatures or in response to shaking in the form of inactive fibrils.^11^ These facts logically initiated efforts to design artificial molecules^12^, ^13^, ^14^, ^15^ that would mimic the action of insulin on IR and could lead to the development of active, metabolically more stable, or even orally available IR agonists. On the other hand, substances capable of inhibiting, either partially or completely, the activation of the receptor by insulin could find application in the control of hypoglycemic states in patients caused by insulin doses that are too high^16^, ^17^ or in preventing problems associated with hyperinsulinemia.^18^

Scientific teams from Novo Nordisk A/S and Antyra, Inc., used the technique of expressing libraries of peptide sequences on the surface of bacteriophage and, by testing their binding affinities, discovered a number of peptides that bound very efficiently to IR while activating or inhibiting it.^19^, ^20^ These peptides also proved to be very useful for studying the mechanism of insulin binding to the receptor, as some of them bound only to binding Site 1, others to binding Site 2 or to both at the same time.^13^ One of these peptides was SLEEEWAQIECEVWGRGCPSY or S592, which bound only to binding Site 2.^6^, ^19^ We show the sequences of peptides referred to in this paper in Table 1 for better orientation to the topic. An interesting feature of peptide S592 is that although it binds to the IR with relatively high binding affinity (*K*_d_ 1.7 × 10^−9^ M), it does not activate the receptor.^19^ Conjugation of peptide S592 to the GSLDESFYDWFERQL peptide, derived from GSLDESFYDWFERQLGKK (S371) that binds only to binding Site 1, using a short flexible linker (GGGSGGS) resulted in peptides S661 and S961 GSLDESFYDWFERQLGGGSGGSSLEEEWAQIQCEVWGRGCPSY with amidated or free C‐terminus, respectively, with native insulin‐like binding affinities for IR and the ability to fully inhibit insulin‐stimulated lipogenesis in cells and the insulin effect on plasma glucose in mice.^21^

**TABLE 1:** Overview of peptide sequences cited

Code	Sequence	Citation
S592	SLEEEWAQIE **C** EVWGRG **C** PSY	^6^, ^19^, ^20^
S371	GSLDESFYDWFERQLGKK	^19^, ^20^
S661	GSLDESFYDWFERQLGGGSGGSSLEEEWAQIQ **C** EVWGRG **C** PSY‐amide	^21^
S961	GSLDESFYDWFERQLGGGSGGSSLEEEWAQIQ **C** EVWGRG **C** PSY	^21^
S519	SLEEEWAQVE **C** EVYGRG **C** PSGSLDESFYDWFERQLG	^13^
Cys → Ser in S661	GSLDESFYDWFERQLGGGSGGSSLEEEWAQIQSEVWGRGSPSY‐amide	^22^
Cys → Ser in S592	SLEEEWAQIQSEVWGRGSPSYC	^22^
peptide **1**	SLEEEWAQIE **C** EVWGRG **C** PS‐amide	This paper
IM459	#SLEQEWaKIE **C** EVYGK **C** PPKKAyKWFERQLK‐amide	^23^
IM172N22	#SLEEEWAQIE **C** EVYGR **C** PPSES‐amide	^23^
S597‐N20	SLEEEWAQIE **C** EVYGRG **C** PS‐amide	^24^

*Note*: The Cys residues forming the disulfide bridge are highlighted in red. # denotes *N*‐terminal phenylacetylation, a means 2‐aminoisobutyric acid, and y is *O*‐methyltyrosine.

**FIGURE 1:**
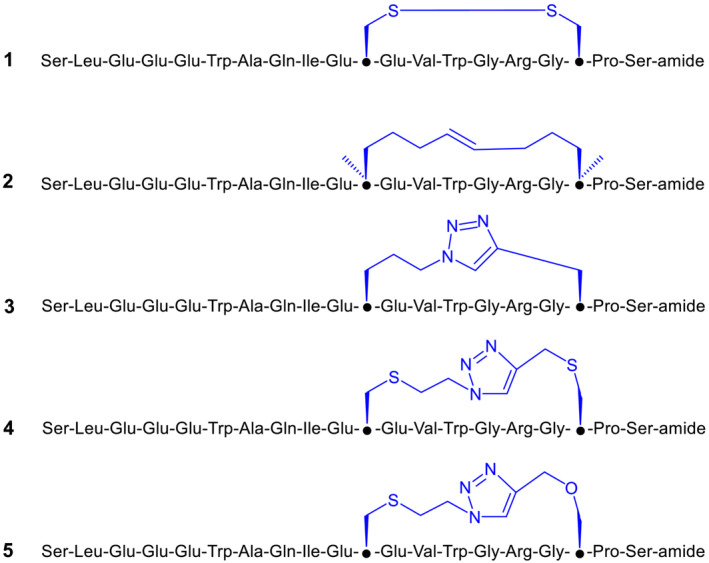
Simplified structures of peptides **1**–**5**. The parts by which the peptides differ are shown in blue. Black dots indicate C^α^ atoms at positions 11 and 18.

In 2018, Brandt et al.^22^ tested the ability of S661 and its variant with cysteines mutated to serines to inhibit insulin‐stimulated IR phosphorylation in cells and observed an eightfold decrease in the antagonistic activity of the variant peptide without the disulfide bridge, indicating the importance of the disulfide bridge in S661 for receptor interaction. They also tested the peptides corresponding to Site 1 (S371) and Site 2 (Cys → Ser in S592) separately and did not detect any agonistic or antagonistic activities. This result inspired us to conduct the present study, in which we prepared five variants of the S592 peptide: peptide **1** with a natural disulfide and peptides **2**–**5** in which we replaced the natural disulfide bridge with 1,2,3‐triazole or dicarba linkages (Figures 1 and S1). The goal of this study was to investigate whether chemical engineering of the cyclization of mimetic peptide **1** can led to modulation of its ability to bind and activate or inhibit IR.

## 2 METHODS

### 2.1 Chemistry

Synthetic protocols, analytical, and NMR data for peptides **1**–**5** and precursor compounds **6**–**24** are provided in the Supporting Information (Figures S2–S17 and Schemes S1–S5).

### 2.2 Receptor‐binding studies

Binding affinities of analogs for IR‐A were determined by the competition of hormones with [^125^I]‐monoiodotyrosyl‐TyrA14‐insulin for IR‐A in cell membranes of human IM‐9 lymphocytes (ATCC) as described previously.^25^, ^26^ Radiolabeled [^125^I]‐monoiodotyrosyl‐TyrA14‐insulin was prepared according to a procedure described in detail by Asai et al.^27^ The binding curve of each analog was determined in duplicate, and the final dissociation constant (*K*_d_) was calculated from at least three binding curves.

### 2.3 Receptor phosphorylation assay and antagonism assay

Mouse embryonic fibroblasts (IR‐A) derived from IGF‐1R knockout mice and stably transfected with human IR‐A, kindly provided by A. Belfiore (Catanzaro, Italy) and R. Baserga (Philadelphia, Pennsylvania, USA), were grown as described previously.^25^ Ligand‐dose response IR‐A autophosphorylation levels for the analogs were determined using an in‐cell Western assay adapted for chemiluminiscence as described in Machackova et al.^28^ Briefly, the IR‐A cells were plated at 20,000 cells/well in white 96‐well Brand plates cell grade (Brand GMBH, Germany) and incubated for 24 h. The cells were starved for 4 h in serum‐free media and stimulated with dilutions of ligands for 20 min. After incubation, the cells were fixed in 3.75% freshly prepared formaldehyde for 20 min. The cells were permeabilized with 0.1% Triton‐X‐100 in PBS for 5 min and blocked with 5% BSA in T‐TBS. Plates were incubated with anti‐phospho‐IGF‐1Rβ (Tyr1135/1136)/IRβ (Tyr1150/1151) overnight at 4°C and developed with peroxidase‐labeled anti‐rabbit secondary antibody (Sigma). SuperSignal West Femto maximum sensitivity substrate was added to each well, and chemiluminescence was detected using the ChemiDoc MP Imaging System. For antagonism, the cells were incubated with the analogs (concentration range from 0.1 nM to 50 μM) in the presence of 10 nM insulin. Data were subtracted from the background values and expressed as the contribution of phosphorylation relative to the 10 nM insulin signal. Each point was measured in duplicate, and the experiment was repeated three times. Control wells and wells stimulated with 10 nM insulin were conducted as tetraplicates on each plate. Nonlinear regression curve fitting of the combined data from all experiments (i.e., six values per point) was carried out with GraphPad Prism 5 software.

Control Western blots were performed as in Krizkova et al.^29^ The IR‐A cells on 24‐well plates were stimulated with 10 μM and 5 μM concentrations of the ligands either alone or in the presence of 10 nM insulin for 10 min. Proteins were routinely analyzed using immunoblotting. The membranes were probed with anti‐phospho‐IGF‐1Rβ (Tyr1135/1136)/IRβ (Tyr1150/1151) (Cell Signaling Technology). Anti‐actin (20–33) antibody (Sigma–Aldrich, cat. A5060) was used as a loading control.

## 3 RESULTS AND DISCUSSION

### 3.1 Synthesis of Fmoc‐protected amino acids **7** and **11**

**SCHEME 1:**
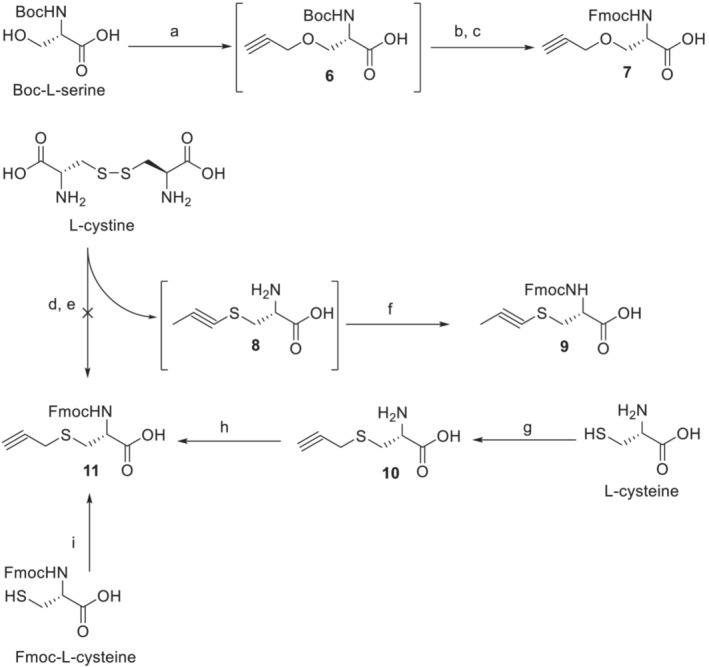
Synthesis of alkyne amino acids **7** and **11**. Reagents, conditions, and yields: (a) NaH, DMF, propargyl bromide, 0°C to room temperature overnight; (b) TFA, DCM from 0°C to room temperature for 2 h; (c) Fmoc‐*O*Su, NaHCO_3_, water, and dioxane, 1 h at 0°C, then overnight at rt (53% yield after three steps); (d) Na, NH_3_(l); (e) propargyl bromide; (f) Fmoc‐*O*Su, NaHCO_3_, water, and dioxane, 1 h at 0°C, then overnight at rt (58% yield after three steps); (g) propargyl bromide, NH_4_OH overnight at rt (56% yield); (h) Fmoc‐*O*Su, NaHCO_3_, water, and dioxane, 1 h at 0°C, then overnight at rt (89% yield); (i) propargyl bromide, NaHCO_3_, TBAB, water, and ethyl acetate, 4 days at rt (77% yield)

The synthesis of Fmoc‐protected amino acids **7** and **11**, precursors of the formation of intramolecular bridges in peptides, is shown in Scheme 1. Compounds **7** and **11** are derived from serine and cysteine and contain propargyl groups connected via O and S heteroatoms, respectively. Since the reactivity of the 2‐propynyl moiety is significantly higher than that of the alkyl moiety, direct alkylation of freshly generated alcoholate or thiolate allowed straightforward syntheses of compounds **7** and **11**. Briefly, treatment of Boc‐L‐serine with sodium hydride produced fully deprotonated amino acids, which were efficiently alkylated with propargyl bromide. Then, the Boc‐protecting group of intermediate **6** was cleaved with TFA, and the free amine was reacted with Fmoc‐*O*Su to produce the final *O*‐derivative **7**. For *S*‐analog **11**, we proceeded in a similar fashion. Free L‐cysteine was transformed to *S*‐propargyl cysteine **10,** and the Fmoc group was added by a reaction with Fmoc‐*O*Su. We also found that compound **11** can be prepared by a one‐step reaction of Fmoc‐L‐cysteine and propargyl bromide under phase transfer conditions with a satisfactory yield (77%). In parallel, L‐cystine was also tested as a potential starting material for the synthesis of **11**. The disulfide bond was reduced by treatment of metallic sodium in liquid ammonia to form a thiolate, and then propargyl bromide was added. Intermediate **8** was easily converted to Fmoc‐protected amino acid **9**. Surprisingly, detailed NMR and infrared spectroscopy analyses of **9** revealed the isomerization of *S*‐(2‐propynyl) to *S*‐(1‐propynyl). We have not found any report on a similar isomerization reaction occurring on alkylated Cys, but the phenomenon of an acetylenic bond shift was described previously.^30^, ^31^ In general, an acetylenic bond shift occurs when a starting material is treated with a strong base in a polar solvent, both protic and aprotic. It is supposed that the shift probably proceeds via allenic intermediates, which have been isolated several times.^32^

### 3.2 Synthesis of Fmoc‐protected amino acid **23**

**SCHEME 2:**
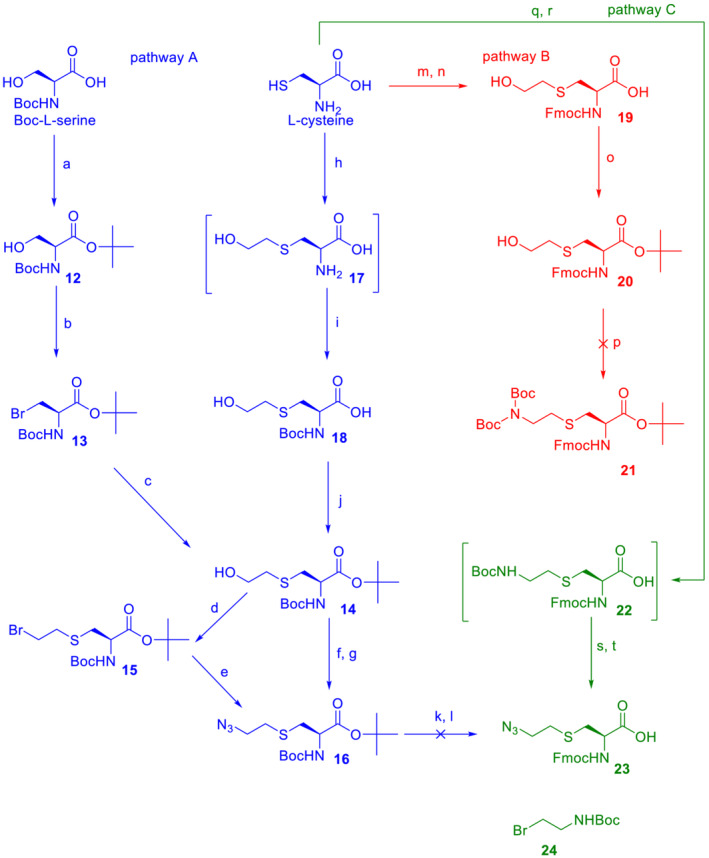
Synthesis of azido amino acid **23**. Reagents, conditions, and yields: *O*‐*tert*‐butyl‐*N*,*N′*‐diisopropyl isourea, DCM, overnight at rt (72% yield); (b) CBr_4_, PPh_3_, DCM, 1 h at 0°C, then overnight at rt (66% yield); (c) HOCH_2_CH_2_SH, K_2_CO_3_, AcCN, overnight at rt (61% yield); (d) CBr_4_, PPh_3_, DCM, 1 h at 0°C, then overnight at rt (68% yield); (e) NaN_3_, DMSO, 70°C overnight (90% yiled); (f) MsCl, TEA, DCM, 1 h at 0°C; (g) NaN_3_, DMSO, 70°C overnight (80% yield for two steps); (h) Na, MeOH, 1 h at rt then BrCH_2_CH_2_OH overnight; (i) Boc_2_O, Na_2_CO_3_, water and dioxane, 0.5 h at 0°C, then overnight at rt (76% yield for two steps); (j) *O*‐*tert*‐butyl‐*N*,*N′*‐diisopropyl isourea, DCM, overnight at rt (55% yield); (k) TFA, TIPS, DCM, 1 h at rt; (l) Fmoc‐*O*Su, NaHCO_3_, water, dioxane, 1 h at 0°C, then overnight at rt; (m) Na, MeOH, 0.5 h at rt then BrCH_2_CH_2_OH 3 h; (n) Fmoc‐*O*Su, NaHCO_3_, water, and dioxane, 1 h at 0°C, then overnight at rt (73% yield for two steps); (o) *O*‐*tert*‐butyl‐*N*,*N′*‐diisopropyl isourea, THF, overnight at rt (77% yield); (p) HN (Boc)_2_, PPh_3_, DIAD, THF overnight at rt; (q) **24**, KOH, MeOH, and THF overnight at rt; (r) Fmoc‐*O*Su, NaHCO_3_, water and dioxane, 0°C then overnight at rt; (s) TFA, TIPS, DCM, 2 h at rt; (t) TfN_3_, NaHCO_3_, methanol, DCM and water, overnight at rt (50% yield for four steps).

The synthesis of Fmoc‐protected azido derivative **23**, a precursor for the formation of intramolecular bridges in peptides, is shown in Scheme 2. Compound **23** could be prepared, for example, by a reaction of Fmoc‐L‐Cys‐OH and 2‐azidoethylbromide under phase transfer conditions. However, handling potentially explosive low molecular azide can be dangerous. Therefore, we decided to add an azido moiety at later stages of the synthesis, when the molecular weight of the compound is higher and the manipulation is safer.

Our first strategy was to prepare fully protected intermediate **16**, which bears an azido moiety and which, after (i) TFA‐mediated cleavage of acidolabile groups and (ii) protection of the free amine with Fmoc, should produce target compound **23** (Pathway A, blue). The carboxylate group of Boc‐L‐serine was chemoselectively esterified, the hydroxy group of **12** was transformed to bromide **13** using the Appel reaction, and finally, the side chain was prolonged with 2‐hydroxyethyl by the nucleophilic reaction of 2‐mercaptoethanol in the presence of a weak base to give **14**.

As we show herein, L‐cysteine also represents a suitable material for the synthesis of ester **14**. Sodium‐generated thiolate of free cysteine was reacted with 2‐bromoethanol, and the amino group was protected by reaction with Boc anhydride to give carboxylic acid **18**, which was easily transformed to an ester by treatment with isourea. To add an azido group, it was necessary to activate the hydroxy moiety of **14** either with bromination or mesylation. Both methods provided compound **16** in high yields. The last step was simultaneous removal of both acidolabile protecting groups of **16** with an acidic cocktail followed by a reaction with Fmoc‐*O*Su. However, after a classical work‐up, we found that the reaction mixture contained at least four main products, none of which corresponded to compound **23**, and none of them had an azido group. Based on these findings, we suppose that the azido group of the (2‐azidoethyl)‐*S*‐cysteine motif can undergo acid‐catalyzed cleavage.

To overcome this difficulty, a complete change of a synthetic strategy was necessary. In Pathways B and C (Scheme 1, in red and green, respectively), the α‐amino group is protected by acid‐stable Fmoc almost from the beginning of the synthesis, and then another primary amino group is added to the side chain, which represents a core for a “future” azide (i.e., a strategy of a “masked” azide). This arrangement makes it possible to use strongly acidic TFA during synthesis, and the last step of the synthesis consists of the transformation of the amino group to an azide that is realized under mild conditions. Thus, the L‐cysteine side chain was prolonged with a 2‐hydroxyethyl moiety and *N*‐protected with Fmoc to give acid **19**, which was esterified with isourea. However, the Mitsunobu reaction of **20** with (Boc)_2_NH performed using standard conditions failed, and product **21** was not obtained (Pathway B).

Therefore, the methodology was modified again (Pathway C), and the amino group was added to the side chain of cysteine at the beginning of synthesis. Briefly, L‐cysteine was reacted with **24** under basic conditions, and the free α‐amine was protected in situ with Fmoc‐*O*Su. Isolated intermediate **22** was treated with TFA, and then the free ω‐amine was subjected to an azido‐transfer reaction to provide the final product **23**. The whole synthesis of **23** in Pathway C consisted of 4 steps and gave a satisfactory cumulative yield of 50%.

### 3.3 Synthesis and NMR spectroscopy of the target stapled peptides **1**–**5**

We synthesized peptides **1**–**5** (Figures 1 and S1), derivatives of the insulin mimetic peptide S592 (Table 1). Peptide **1**, similar to S592, has a natural disulfide bridge connecting positions 11 and 18. Peptides **2**–**5** differ from peptide **1** in that the original disulfide bridge (four atoms) between positions 11 and 18 is replaced by other chemical *intra* chain tethers. The stereochemistry at positions 11 and 18 has not been changed. Peptide **2** has an 8‐atom “dicarba” staple with a double bond and additional methyl groups on the C^α^ atoms of the respective amino acids. We have recently used the same kind of staple linker to stabilize the secondary structure of preptin fragments.^33^ Peptides **3**–**5** were prepared by Cu^(I)^‐catalyzed cycloaddition of different azido and alkyne amino acid precursors following the protocol we described earlier^33^ at position 11 and alkyne precursors at position 18. Peptide **3** with a triazole‐containing 7‐atom long staple was prepared by “clicking” δ‐azido‐pentanoic acid^34^ at 11 and propargyl glycine at 18. Peptides **4** and **5** have longer triazole‐containing 10‐atom staples, and they differ from **3** by the presence of sulfur (in **4**) or sulfur and oxygen (in **5**) atoms in the staples. The synthesis of azido and alkyne precursors is described in the Methods and Supporting Information.

The structures of the stapled peptides **1–5** were assessed by measuring the ^1^H NMR spectra. The proton signals of peptides **1–3** showed remarkable line broadening at 25°C. This can arise from a medium rate of conformation time averaging. The complete structure assignment of peptides **1** and **2** was, therefore, achieved in the NMR spectra measured at 45°C, when peptide **1** showed dramatic signal narrowing, allowing for the observation of *J* (NH,Hα) splitting of NH signals. The NMR spectra of peptide **2** showed a doubling of some signals as the result of *cis‐* and *trans*‐isomers present in this sample. The attempts to separate them were not successful. Proton NMR data for peptides **1–5** are summarized in Tables S1–S5. The chemical shifts of their backbone Hα protons (see Figure S16) are similar, with larger differences mainly for structurally different residues in positions **11** and **18**. The absence of characteristic medium‐ or long‐range contacts in the 2D‐H,H‐NOESY spectra together with the abovementioned line broadening indicate the considerable flexibility of peptides **1–5**. When applying the chemical shift index (CSI method, for details, see Wishart and Sykes^35^) to Hα‐shifts, some population of helical structures can be expected, but the observed negative CSI values are often smaller than the characteristic value of 0.1 ppm. The CSI diagrams of peptides **1** and **2** (Figure S17) indicated a higher population of helical conformations in their structures. Some support for the contribution of helical forms can also be deduced from *J* (NH,Hα) obtained for peptides **1** and **5** (Tables S1 and S5), which are generally lower than their random‐coil values (~8 Hz) but mostly still higher than the values <5 Hz typical for helices. Overall, the results of the NMR measurements suggest that the structures of peptides **1**–**5** are flexible. Indications of the presence of helical structures for some peptides (e.g., **1** and **2**) in solution are not significant.

### 3.4 Binding affinities of peptides **1**–**5** to IR‐A and their abilities to stimulate phosphorylation of this receptor

First, the binding affinities of peptides **1**–**5** to insulin receptor isoform A (IR‐A) were measured. The *K*_d_ values are shown in Table 2, and representative binding curves are shown in Figure S18. Peptide **1** binds to IR‐A with 3.9 × 10^−9^ binding affinity, which makes it only approximately 13 times weaker than native insulin (Table 2 and Figure S2). This is a remarkably high affinity, considering that **1** has only 20 amino acids (insulin has 51 amino acids) and is supposed to bind only to Site 2 in the IR.

**TABLE 2:** Binding affinity for IR‐A and ability to stimulate IR‐A autophosphorylation by human insulin and peptides **1–5**

Peptide	Type of the intramolecular staple (number of atoms in the staple)	*K*_d_ ± S.D. (nM) for binding to IR‐A (*n* ≥ 3)	EC_50_ (nM) (best fit values) for stimulation (S) or antagonism (A)	EC_50_/*K*_d_
Insulin		0.31 ± 0.02	3.0 (S)	10
**1**	disulfide (4)	3.9 ± 1.0	7.2 (A)	1.8
**2**	dicarba (8)	49 ± 6	274 (A)	5.6
**3**	triazole (7)	15 ± 3	92 (A)	6.1
**4**	triazole‐sulfur‐sulfur (10)	25 ± 7	28 (A)	1.1
**5**	triazole‐sulfur‐oxygen (10)	75 ± 29	389 (A)	5.5

*Note*: *K*_d_ values for receptor binding were obtained from at least three measurements. EC_50_ values for stimulation of IR‐A receptor autophosphorylation by insulin or for inhibition (antagonism) of insulin‐stimulated autophosphorylation by peptides **1**–**5** were obtained from the best fit of the curves shown in Figure 2. Data are expressed as the contribution of phosphorylation relative to the human insulin signal at 10 nM. Details are given in Section 2 and representative binding curves are shown in Figure S18.

All new mimetic peptides **2**–**4** also bound the receptor with nanomolar affinity but were weaker IR‐A binders than peptide **1**. Considering the length of the staple, that is, the number of atoms in the linker bridging positions 11 and 18 in the peptides, a shorter staple seems to be clearly preferable for improving the binding affinity. After peptide **1** with four atoms, the second strongest binder is peptide **3** with seven atoms. Interestingly, derivative **4**, with a 10‐atom long linker, is stronger in binding than dicarba analog **2**, with eight atoms. It is possible that the negative effect of the longer linker is balanced by the positive effect of the heteroatoms in the linker. On the other hand, the difference in binding affinities of analogs **4** and **5**, which have equally long linkers, seems to be due to the negative effect of the oxygen atom in **5**, which is less favorable than the sulfur in **4**.

**FIGURE 2:**
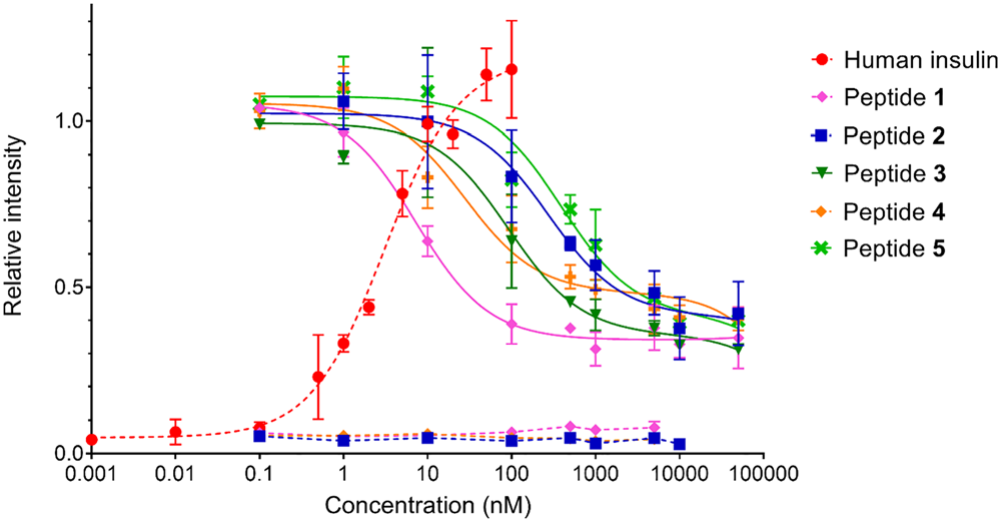
Relative abilities of peptides **1**–**5** to antagonize insulin‐stimulated receptor phosphorylation (full lines). IR‐A transfected cells were stimulated with 0.0001 μM to 50 μM peptides in the presence of 10 nM insulin. Dashed lines show stimulation of IR‐A with insulin and selected peptides **1**, **2**, and **4** alone. The data are expressed as the contribution of phosphorylation relative to the signal of human insulin at 10 nM and are shown with S.D. The details are provided in Section 2.

A remarkable feature of peptides **1**–**5** is that, unlike insulin, they do not stimulate receptor autophosphorylation over a wide range of concentrations (dashed lines in Figures 2 and S19). In addition, these compounds antagonize (inhibit) the ability of insulin to stimulate receptor autophosphorylation (full lines in Figure 2). Interestingly, their antagonism appears to be only partial, as the receptor retains approximately 30% of its activity even at the highest concentrations tested of peptides **1**–**5**.

This result is in agreement with previous findings that only fusion peptides (such as S661 and S961) binding both Site 1 and Site 2 on the receptor can behave as full agonists or antagonists.^13^ The Site 2 peptide with the Cys‐to‐Ser mutations (see above, Table 1) produced by Brandt et al.^22^ was able to lower the 1 nM insulin signal to only 50–60% (at the concentration 100 nM) compared with our peptides **1**–**5**, which were able to block 10 nM insulin signaling up to 30%.

Another argument supporting the important role of the cyclized part of the mimetics (or their sulfur atoms) is the low binding affinity of the SLEEEWAQ‐amide peptide (900 nM)^36^ compared with peptides **1**–**5** (4–75 nM). These data suggest that the disulfide bridge in the “Site 2 part” of mimetics, such as the S661 peptide, is important for IR binding and also for the ability of the peptides to inhibit insulin‐induced IR stimulation. This can be supported by the observation that our mimetic peptides, which differ in their cyclization staples, do not inhibit insulin‐stimulated IR activation in proportion to their binding affinity for IR. The right column in Table 1 reflects these differences between peptides **1** and **5**. While the relative ratio of the peptides' ability to antagonize the action of insulin and binding affinity for IR (EC_50_/*K*_d_) is approximately 1–2 for peptides **1** and **4**, the values for peptides **2**, **3**, and **5** are higher (5–6). This could indicate that the ability of peptides **2**, **3** and **5** to antagonize IR is disproportionate to their binding affinity and weaker than that of peptides **1** and **4**. Interestingly, both peptides **1** and **4** have sulfur atoms in the same positions in their cyclization linkers, but peptides **2** and **3** do not have sulfur atoms, and **5** has one sulfur replaced by oxygen.

Schaffer et al.^13^ hypothesized that mimetic peptides such as S592 (Table 1) containing a SLEEEWAQ segment and a C‐terminal disulfide‐linked moiety should bind to the predicted Site 2 in the IR. On the other hand, mimetic peptides such as S371 (Table 1) should be specific for putative Site 1 in the IR. This hypothesis was later confirmed by Lawrence and coauthors,^37^ who reported the three‐dimensional crystal structure of a 16‐residue fragment of the mimetic peptide S371 (GSLDESFYDWFERQLG) in complex with the L1 and CR domains of the IR. The structure showed that the peptide fragment (SLDESFYDWFERQL) binds to a similar site on the L1 surface as the critical α‐CT IR peptide. Thus, the mimetic peptide disrupts primary Site 1 of the IR.

Site 2 in the IR has been defined by structural studies^3^, ^4^ and is thought to be located on the membrane proximal part of the FnIII‐1^(‘)^ domain in the IR. Nielsen et al.^6^ proposed that the first contact of insulin with the apo IR is mediated by insulin binding specifically to Site 2, followed by insulin translocation (or binding of an additional insulin molecule) to Site 1 on the IR formed by the L1 domain, the α‐CT′ peptide, and the membrane distal segment of the FnIII‐1′ domain. These events are accompanied by global structural changes in the receptor that lead to close contact between the two tyrosine kinase domains and receptor activation. Thus, the cooperation of both Sites 1 and 2 and their translocation is required for full receptor activation.

This hypothesis was recently supported by the resolution of an “asymmetric” cryo‐EM structure of IR with an insulin derivative bound simultaneously at both Sites 1 and 2.^38^ This structure could represent a snapshot of insulin translocation from Site 2 to Site 1. Another important clue supporting the model of Nielsen et al. was published by Wu et al.,^17^ who showed that insulin dimers linked via their B29 residues, which behave as partial agonists of the IR, bind simultaneously to both Site 1 and Site 2, and this “cross‐linking” does not allow complete structural transition and receptor activation. In light of these findings, it seems likely that peptides such as S661 and S961 (Table 1) bind simultaneously to both Sites 1 and 2 and that the short GGSGGSS linker does not allow separation of the two sites, resulting in fully antagonistic effects of the peptides.

Interestingly, swapping the Site 1 and Site 2 peptide sequences in S661 or S961 provided S519 (Table 1), which bound to IR with very high affinity,^13^ similar to S661 and S961,^21^ but it did not show antagonistic effects and instead agonized IR. Recently, Kirk et al.^23^ published a cryo‐EM structure of the human IR ectodomain in complex with the peptide IM459 (Table 1), which is a variant of S519 with several modifications that increase its binding affinity and it retains the ability to activate IR (agonist). They showed that IM459 cross‐links IR domain L1 to FnIII‐1′ and, in doing so, releases constraints on the membrane proximal parts of IR, which allows full activation of the receptor. They were also able to determine the 2.9 Å cryo‐EM structure of the IM172N22 peptide (Table 1), representing “the Site 2 part” of IM459 when the complex was stabilized with prebound insulin at Site 1 formed by L1 and the membrane distant part of FnIII‐1′. The salient feature was that INMLN22 engaged the same FnIII‐1′ surface (membrane proximal part) as that observed for the Site 2 insulin in the four‐insulin‐bound IR structure, confirming previous presumptions that IM172N22 and related sequences, such as our peptides, are Site 2 binders. Another important aspect of IM172N22 binding was that its residues Glu3 to Val13 adopt a mostly α‐helical conformation. The interactions with IR were mediated mainly by the Leu2, Glu3, Glu5, Trp6, Ile9, Glu12, and Val13 side chains, and the residues Ser1, Glu4, Ala7, Gln8, Glu10, Cys11, Arg16, Cys17, Pro18, and Pro19 were devoid of interactions with IR. This shows that the interaction is mediated by the *N*‐terminal helical part of the peptide and, importantly, with the disulfide bridge exposed to solvent. The observation of the bound helical conformation of IM172N22 contrasts with our NMR measurements, which indicate rather flexible conformations of our peptides. However, the differences in the binding affinities of peptides **1**–**5** suggest that the type of bridge is important for potent binding. It is possible that helical structures in peptides **1**–**5** can be (at least partly) induced by a chaperoning‐like effect of the receptor upon binding. The similar binding affinities of peptide **1** (3.9 × 10^−9^ M) and peptide IM172N22 (3.1 × 10^−9^ M) support this hypothesis. Thus, the differences in the affinities of our peptides may be due to the ability of individual *intra* chain bridges to modulate the secondary structure of the peptides.

Another paper relevant to our study was recently published by Park et al.,^24^ who were able to solve the structure (3.5 Å) of the mouse IR in complex with the S597N22 peptide (Table 1), which is almost identical to our peptide **1**. Remarkably, the structure of the receptor with the S597N22 peptide bound to the FnIII‐1 domain (Site 2) was similar to the structure of apo‐IR (7SL1).^5^ This finding further supports the hypothesis that Site 2 peptides cannot induce the conformational change required for signaling.

The mechanism by which S592 and its derivatives presented in this study partially inhibit IR is not fully clear. It seems likely that the peptides bind similarly to Site 2 of the receptor as IM172N22 or S597N22 and that they can compete with insulin for Site 2 and are able to displace it effectively from binding. However, binding to Site 2 alone is apparently not sufficient to trigger a structural change in the IR that could lead to its activation, possibly due to its inability to reduce the known conformational flexibility of the apo‐IR.^3^, ^39^ This hypothesis is supported by unsuccessful attempts to obtain the cryo‐EM structure of IR in complex only with IM172N22 by Kirk et al.^23^ and by the inactive apo‐like IR structure bound to S597N22 peptide by Park et al.^24^ On the other hand, the complex of IR with simultaneously bound mimetic peptide in Site 2 and insulin in Site 1^23^ shows that binding of the mimetic peptide to Site 2 is unlikely to completely prevent insulin binding to Site 1, and this could be the mechanism of the only partial antagonism of S592 and its derivatives in the presence of insulin, where even at the highest peptide concentrations, the receptor is still active at approximately 30% of its maximal activity. It is also possible that the different chemical staples in our cyclic peptides may not only affect the binding of the peptide to Site 2 but may also interfere differently with the binding of insulin to vacant Site 1, for example, by imposing less or more structural constraints on the insulin molecule, which may result in their disproportionate binding‐activating properties.

## 4 CONCLUSIONS

In this work, we prepared five variants of a 20 amino acid insulin‐mimicking peptide to specifically target Site 2 of the insulin receptor. These peptides differ from each other by the structure of the covalent bridge connecting positions 11 and 18. In addition to the peptide with a natural disulfide bridge (**1**), a derivative with a dicarba bridge (**2**) and three derivatives (**3**–**5**) with a 1,2,3‐triazole differing from each other by the presence of sulfur or oxygen in their structures were prepared. The strongest binding to IR was exhibited by peptide **1** with a natural disulfide bridge whose affinity was only approximately 13 times lower than that of human insulin. All other derivatives bound to IR approximately 4–19 times more weakly than **1**, and a relationship between increasing connecting bridge length and lower binding affinity can be inferred. Despite their nanomolar binding affinities, none of the prepared peptide mimetics was able to activate the insulin receptor even at high concentrations, but all of the mimetics were able to inhibit insulin‐induced receptor activation. However, the receptor remained approximately 30% active even at the highest concentration of the peptides; thus, they behave as partial antagonists. An interesting observation is that mimetic peptides do not antagonize insulin action in proportion to their binding affinities. Apparently, the presence of sulfur atoms in the cyclization bridge is advantageous both for improving their binding affinity and for inducing the antagonistic effect of the compounds. On the other hand, the absence of sulfur in the linker reduces the affinity of the substances and, to an even greater extent, their ability to antagonize insulin. The compounds characterized in this study show that it is possible to modulate the functional properties of insulin receptor peptide ligands using unnatural disulfide bridge mimetics. We believe that detailed knowledge of how mimetic peptides interact with IR binding sites 1 and 2 may lead to the development of effective nonpeptide insulin mimetics in the future and that this study is a step toward this goal.

## Supporting information


**Figure S1.** Structures of peptides **1**–**5**. The parts by which the peptides differ are in blue.
**Figure S2.** HPLC profile of purified compound **7** using a gradient from Method 1.
**Figure S3.** HPLC profile of purified compound **9** using a gradient from Method 1.
**Figure S4.** HPLC profile of purified compound **11** (prepared from compound **10**) using a gradient from Method 1.
**Figure S5.** HPLC profile of purified compound **11** (prepared Fmoc‐L‐Cys‐OH) using a gradient from Method 1.
**Figure S6.** HPLC profile of purified compound **19** using a gradient from Method 1.
**Figure S7.** HPLC profile of crude compound **22** using a gradient from Method 1.
**Figure S8.** RP‐HPLC profile of purified compound **23** using a gradient from Method 1.
**Figure S9.** Spyder Mark IV Multiple Peptide Synthesizer (http://dc.uochb.cz/index.php).
Figure **S10**. Analytical HPLC profiles of purified peptides **1–5**.
**Figure S11.** Mass spectrum of peptide **1**. Exact MH + expected 2305.0 (C_99_H_145_N_27_O_33_S_2_).
**Figure S12.** Mass spectrum of peptide **2**. Exact MH + expected 2351.2 (C_107_H_159_N_27_O_33_).
**Figure S13.** Mass spectrum of peptide **3**. Exact MH + expected 2336.1 (C_103_H_150_N_30_O_33_).
**Figure S14.** Mass spectrum of peptide **4**. Exact MH + expected 2414.1 (C_104_H_152_N_30_O_33_S_2_).
**Figure S15.** Mass spectrum of peptide **5**. Exact MH + expected 2398.1 (C_104_H_152_N_30_O_34_S).
**Figure S16.** Comparison of Hα chemical shifts in peptides **1–5.**
**Figure S17**. The Δδ (Hα) values (left) and corresponding CSI diagrams of peptides **1**–**5** (right).
**Figure S18.** Representative binding curves of human insulin and peptides **1**–**5** on IR‐A.
**Figure S19.** Representative Western blots for the abilities of peptides to stimulate IR‐A phosphorylation and to antagonize insulin‐stimulated IR‐A phosphorylation. Cells were stimulated with 10 μM and 5 μM ligands alone, or in the presence of 10 nM insulin for 10 min. Control is no stimulation, Ins is 10 nM insulin. Each analog was tested in 4 wells as denoted by the line as 10 μM, 5 μM, 10 μM + Ins, 5 μM + Ins. Membranes were cut at 75 kDa and 50 kDa standards, and respective parts were developed with anti‐phospho‐IGF‐1Rβ (Tyr1135/1136)/IRβ (Tyr1150/1151) antibody (Mr above 75 kDa) and with anti‐actin antibody (Mr below 50 kDa).
**Scheme S1**. Preparation peptide **1**.
**Scheme S2**. Synthetic scheme of preparation peptide **2**. o denotes Cα atom of non‐standard amino acids.
**Scheme S3**. Preparation peptide **3**. o denotes Cα atom of non‐standard amino acids.
**Scheme S4**. Preparation peptide **4**. o denotes Cα atom of non‐standard amino acids.
**Scheme S5**. Preparation peptide **5.** o denotes Cα atom of non‐standard amino acids.
**Table S1**. Proton NMR data of peptide **1** (600 MHz; in H2O + D2O 95:5 + AcOD; pH = 3.0; T = 45°C)
**Table S2**. Proton NMR data of peptide **2** (600 MHz; in H2O + D2O 95:5 + AcOD; pH = 3.0; T = 45°C)
**Table S3.** Proton NMR data of peptide **3** (600 MHz; in H2O + D2O 95:5 + AcOD; pH = 3.0; T = 25°C)
**Table S4.** Proton NMR data of peptide **4** (600 MHz; in H2O + D2O 95:5 + AcOD; pH = 3.0; T = 25°C)
**Table S5**. Proton NMR data of peptide **5** (600 MHz; in H2O + D2O 95:5 + AcOD; pH = 3.0; T = 25°C)
