# Supplementary material for: Modulation of the antagonistic properties of an insulin mimetic peptide by disulfide bridge modifications
Source: J Pept Sci. 2023 Jan 25;29(7):e3478. doi: 10.1002/psc.3478 (PMC10909431; doi:10.1002/psc.3478)
Supplement: Supplementary file 1 — Figure S1. Structures of peptides 1–5. The parts by which the peptides differ are in blue. Figure S2. HPLC profile of purified compound 7 using a gradient from Method 1. Figure S3. HPLC profile of purified compound 9 using a gradient from Method 1. Figure S4. HPLC profile of purified compound 11 (prepared from compound 10) using a gradient from Method 1. Figure S5. HPLC profile of purified compound 11 (prepared Fmoc‐L‐Cys‐OH) using a gradient from Method 1. Figure S6. HPLC profile of purified compound 19 using a gradient from Method 1. Figure S7. HPLC profile of crude compound 22 using a gradient from Method 1. Figure S8. RP‐HPLC profile of purified compound 23 using a gradient from Method 1. Figure S9. Spyder Mark IV Multiple Peptide Synthesizer (http://dc.uochb.cz/index.php). Figure S10. Analytical HPLC profiles of purified peptides 1–5. Figure S11. Mass spectrum of peptide 1. Exact MH + expected 2305.0 (C99H145N27O33S2). Figure S12. Mass spectrum of peptide 2. Exact MH + expected 2351.2 (C107H159N27O33). Figure S13. Mass spectrum of peptide 3. Exact MH + expected 2336.1 (C103H150N30O33). Figure S14. Mass spectrum of peptide 4. Exact MH + expected 2414.1 (C104H152N30O33S2). Figure S15. Mass spectrum of peptide 5. Exact MH + expected 2398.1 (C104H152N30O34S). Figure S16. Comparison of Hα chemical shifts in peptides 1–5. Figure S17. The Δδ (Hα) values (left) and corresponding CSI diagrams of peptides 1–5 (right). Figure S18. Representative binding curves of human insulin and peptides 1–5 on IR‐A. Figure S19. Representative Western blots for the abilities of peptides to stimulate IR‐A phosphorylation and to antagonize insulin‐stimulated IR‐A phosphorylation. Cells were stimulated with 10 μM and 5 μM ligands alone, or in the presence of 10 nM insulin for 10 min. Control is no stimulation, Ins is 10 nM insulin. Each analog was tested in 4 wells as denoted by the line as 10 μM, 5 μM, 10 μM + Ins, 5 μM + Ins. Membranes were cut at 75 kDa and 50 kDa standards, and respect [file PSC-29-e3478-s001.pdf]

SUPPORTING INFORMATION

for

Modulation of the antagonistic properties of an insulin mimetic peptide by disulfide bridge modifications

*Marta Lubos[#], Jan Pícha[#], Irena Selicharová, Jiří Žák, Miloš Buděšínský, Katarína Mitrová, Lenka Žáková and Jiří Jiráček**

[#] These two authors contributed equally to this work. * Correspondence to jiracek@uochb.cas.cz.

Table of contents:

List of abbreviations

Detailed structures of peptides ***1-5*** *(Figure S1)*

Synthesis of precursor compounds ***6*** *–* ***24***

Analytical chromatograms of compounds ***7****,* ***9****,* ***11****,* ***19****,* ***22*** *and* ***23*** *(Figures S2-S8)*

Solid-phase peptide synthesis of peptides ***1-5*** *(Figure S9)*

Formation of disulfide bridge in peptide ***1*** *(Scheme S1)*

Staple formation by ring-closing olefin metathesis (RCM) in peptide ***2*** *(Scheme S2)*

$Cu^{(I)}$-catalyzed azide-alkyne cycloaddition (CuAAC, click reaction) of peptides ***3-5*** *(Schemes S3-S5)*

Analytical chromatograms of peptides ***1-5*** *(Figures S10-S15)*

NMR data of peptides ***1-5*** *(Tables S1-S5, Figures S16 and S17)*

Representative binding curves of peptides ***1-5*** *and human insulin for IR-A (Figure S18)*

Representative Western blots for the abilities of peptides to stimulate IR-A phosphorylation and antagonize insulin-stimulated IR-A phosphorylation (Figure S19)

Supplementary references

List of abbreviations

Boc	*tert*-butyloxycarbonyl
Boc_2O	di-*tert*-butyl dicarbonate
CuAAC	copper-catalyzed azide-alkyne cycloaddition
DCM	dichloromethane
DIC	*N*, *N´*-diisopropylcarbodiimide
DIU	*N*, *N´*-diisopropylurea
DIPEA	*N*, *N* - diisopropylethylamine
DMF	dimethylformamide
DMSO	dimethyl sulfoxide
Fmoc	9-fluorenylmethyloxycarbonyl
Fmoc-*O*Su	*N*-(9-fluorenylmethoxycarbonyloxy)succinimide
HBTU	1-[bis(dimethylamino)methylene]-1*H*-benzotriazolium 3-oxide hexafluorophosphate
HOBT	1-hydroxybenzotriazole
IPA	isopropyl alcohol
MsCl	methanesulfonyl chloride
RCM	ring-closing olefin metathesis
RP-HPLC	reverse-phase HPLC
SPPS	solid-phase peptide synthesis
TEA	triethylamine
TFA	trifluoroacetic acid
TLC	thin-layer chromatography
TIPS	triisopropyl silane
Trt	Trityl

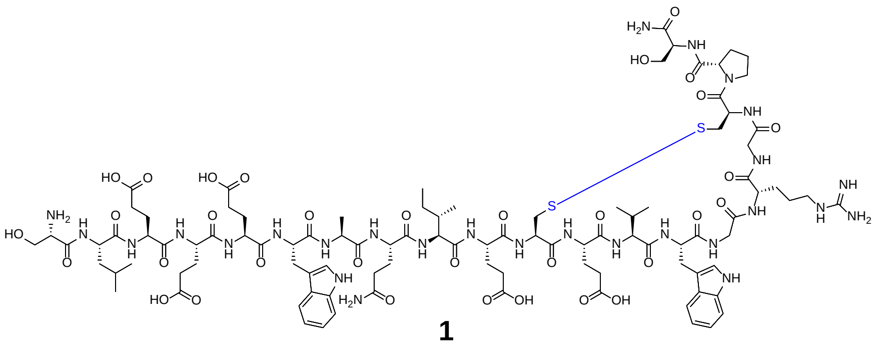

1

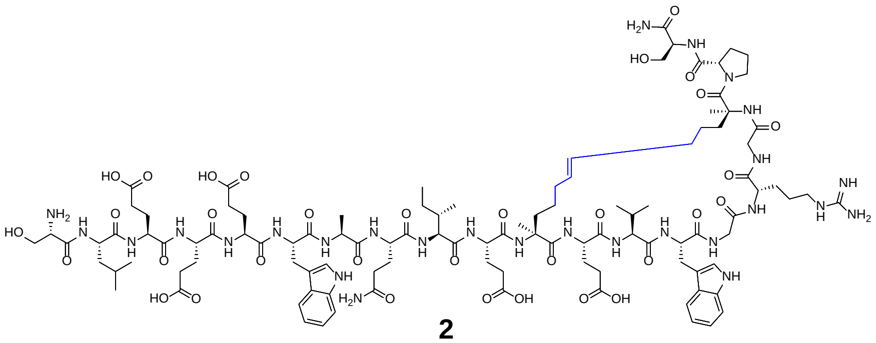

2

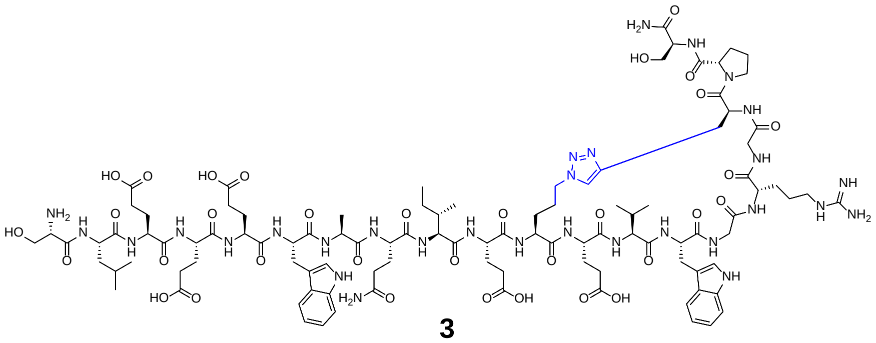

3

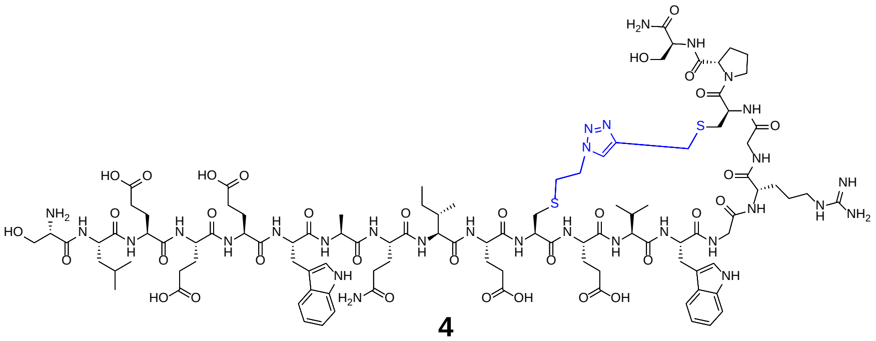

4

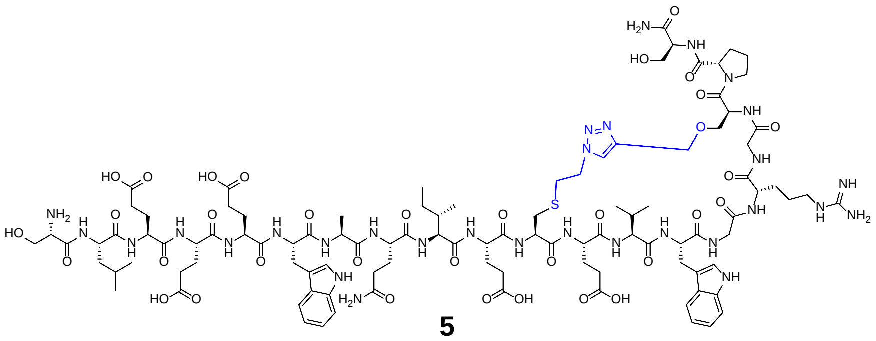

5

Figure S1. Structures of peptides **1-5**. The parts by which the peptides differ are in blue.

Synthesis of precursor compounds ***6 – 24***

General information

Unless otherwise stated, solvents used in this study were obtained from commercial suppliers (Penta chemicals, Lach-Ner, VWR Chemicals). Boc-L-serine, L-cysteine, L-cystine, 2-bromoethanol and *N*-Boc-ethanolamine were purchased from Fluorochem Ltd. TLC was performed on silica gel-coated aluminum plates (Merck). The compounds were visualized by exposure to UV light at 254 nm, by ninhydrin spraying followed by heating (dark blue color of primary amines), by 1 % $KMnO_4$ spraying (yellow color of oxidized products of thioethers or triple bond). Flash chromatography was carried out on silica gel (40-63 μm, VWR Chemicals). Analytical RP-HPLC was carried out with solvent A (0.1 % v/v TFA in water and solvent B (80 % v/v acetonitrile in 0.1 % v/v TFA in water) using Method 1 gradient: t = 0 min (20 % B), t = 30 min (100 % B), t = 31 min (20 % B) at a flow rate of 1 ml/min on a C8 column (EC 250-4.6 Nucleosil 100-5 C8, Macherey-Nagel). Preparative RP-HPLC was carried out with the same solvents using Method 2 gradient: t = 0 min (50 % B), t = 30 min (100 % B), t = 31 min (50 % B) on a C4 column (Grace Vydac C4 214TP; 250×22 mm, 10–15 μm) at a flow rate of 9 ml/min. The eluted compounds were detected at 218 nm.

Melting points were determined on a Boetius block and are uncorrected.

Optical rotation values were measured in 100 mm cell on Perkin Elmer 241 MC under Na lamp radiation.

^{1}H and ^{13}C NMR spectra were acquired on Bruker AVANCE-600 spectrometer (^{1}H at 600.13 MHz, ^{13}C at 150.9 MHz) in $CDCl_3$ or DMSO-d_6 at 300 K. The 2D-H,H-COSY, 2D-H,C-HSQC and 2D-H,C-HMBC spectra were recorded and used for the structural assignment of proton and carbon signals. IR spectra were recorded on a Bruker IFS 55 Equinox apparatus.

HRMS data were obtained on a FTMS mass spectrometer LTQ-orbitrap XL (Thermo Fisher, Bremen, Germany) in electrospray ionization mode.

(*S*)-2-[(*tert*. butoxycarbonyl)amino]-3-(prop-2-yn-1-yloxy)propanoic acid **6**

Compound **6** was prepared using slightly modified protocol published by T. J. Sminia [1]. Boc-L-Ser-OH (5 g; 24.4 mmol) was dissolved in 80 ml of DMF and 60 % NaH (2.4 g; 60 mmol) was carefully

(severe liberation of hydrogen) added in several portions during ice-cooling. After stirring at 0 °C for 30 minutes, 80 % propargyl bromide (3.3 ml; 30 mmol) was added dropwise to the resulting white slurry. The cooling bath was removed, and the reaction mixture was allowed to react overnight at rt. Next, the mixture was again ice-cooled, 100 ml water was added and then the pH value was adjusted approximately to 3 with 10 % citric acid. A brown solution was extracted 3 x with 100 ml ethyl acetate; the combined organic layers were washed 2 x with 100 ml brine, dried with Na_2SO_4 and concentrated under reduced pressure. The resulting oil (8 g) was subjected to flash chromatography on silica, using a linear gradient of ethyl acetate in toluene to afford 4.85 g (*S*)-2-[(*tert*. butoxycarbonyl)amino]-3-(prop-2-yn-1-yloxy)propanoic acid **6** (yellow oil). The purity of **6** was checked by TLC chromatography; R_f = 0.52 (ethyl acetate : MeOH : acetone : water = 4 : 1 : 1 : 1, plate was exposed before elution by gaseous ammonia).

(*S*)-2-{[((Fluorenyl-9-yl)methoxy)carbonyl]amino}-3-(prop-2-yn-1-yloxy)propanoic acid **7** (Fmoc-L-Ser(Prp)-OH)

Intermediate **6** (4.85 g; 19.95 mmol) was dissolved in 5 ml of DCM, and 10 ml of TFA was added during ice-cooling. The reaction mixture was stirred for another 2 hours at rt and the complete removal of Boc group was checked by TLC analysis (ethyl acetate : MeOH : acetone water = 6 : 1 : 1 : 0.5). Volatile solvents were evaporated and the oil brown residue was dissolved in 50 ml of saturated aqueous solution of $NaHCO_3$, ice-cooled, and Fmoc-*O*Su (6.7 g; 19.95 mmol) in 50 ml dioxane was added dropwise. The reaction mixture was stirred for 1 hour at 0 °C and then overnight at rt. The flask was cooled again in an ice bath and 1M HCl was added until pH ~ 1 was achieved. The reaction mixture was extracted 3 x with 100 ml ethyl acetate; the combined organic phases were washed with 100 ml water, 100 ml brine, dried on Na_2SO_4 and concentrated *in vacuo* to give a crude solid. Double crystallization from a mixture of ethyl acetate and hexane afforded the compound **7**. Yield 4.7 g (53 %) for 3 steps. Beige solid, m. p. 130 – 132 °C. R_f = 0.69 (ethyl acetate : MeOH : acetone water = 6 : 1 : 1 : 0.5). $[\alpha]_D^{20}$ = -3.7 (c = 2 ; DMF); ^{1}H NMR (600 MHz, $CDCl_3$; T = 25 °C): 3.46 t (1H, *J* = 2.4 Hz, ≡CH), 3.72 m (2H, −CH₂−O), 4.16 d (2H, *J* = 2.4 Hz, −CH₂−O), 4.22 m (1H, >CH−N), 4.23 m (1H,

>CH−), 4.28 m (2H, −CH_2−O), 7.33 td (2H, *J* = 7.5 and 1.0 Hz, 2x ArH (Fmoc)), 7.42 td (2H, *J* = 7.5 and 1.0 Hz, 2x ArH (Fmoc)), 7.65 br d (1H, *J* = 8.0 Hz, >NH), 7.74 dt (2H, *J* = 7.5 and 1.0 Hz, 2x ArH (Fmoc)), 7.89 dt (2H, *J* = 7.5 and 1.0 Hz, 2x ArH (Fmoc)). ^{13}C NMR (150.9 MHz, $CDCl_3$; T = 25 °C): 46.78 (>CH− (Fmoc)), 54.18 (>CH−N), 57.85 (−CH_2−O), 65.96 (−CH_2−O (Fmoc)), 68.88 (−CH_2−O), 77.70 (≡CH), 80.06 (≡C−), 120.28 (2x =CH−(Fmoc)), 125.50 (=CH−(Fmoc)), 125.53 (=CH−(Fmoc)), 127.26 (2x =CH−(Fmoc)), 127.82 (2x =CH−(Fmoc)), 140.88 (2x =C< (Fmoc)), 143.98 (2x =C< (Fmoc)), 156.20 (O−CO−N), 171.66 (−COOH). IR (KBr, ν_{max} cm^{-1}) 3263 s (≡C−H); 2113 w (C≡C); 1762 s (C=O) acid, 1723 vs (C=O) carba; 1526 s (amide II); 3404 s (NH); 3066 m, 1612 m, 1580 w, 1477 m, 1451 m, 1109 s, 1035 s, 757 m, 739 s, 621 m (fluorene). HRMS (ESI) calculated for $C_{21}H_{18}O_5N$ $[M-H]^-$ 364.11905; found: 364.11895.

(*R*)-2-{[((Fluorenyl-9-yl)methoxy)carbonyl]amino}-3-(prop-1-yn-1-ylthio)propanoic acid **9**

L-Cystine (4.8 g; 20 mmol) was loaded into a 500 ml Erlenmeyer flask, fitted with a Claisen adapter, a dry ice cooler, a glass-coated magnetic rod, and potassium hydroxide tubing at the inlet and outlet. The apparatus was first flushed with a stream of dry ammonia. When approximately 200 ml of liquid ammonia was condensed, the gas intake was closed, the inlet tube was removed and replaced with a stopper. Then, metallic sodium (1.93 g; 84 mmol) was added in several small portions during 1 h until the dark blue color persisted for at least half an hour. The excess of free sodium was quenched by adding a small portion of ammonium chloride, which resulted in full decolorization of the solution. Subsequently, 80 % propargyl bromide (4.7 ml; 40 mmol) was added, stirring and cooling was stopped, and ammonia was allowed to evaporate spontaneously overnight. The resulting dark solid was dissolved in 100 ml water and 1 M HCl was added during ice-cooling and stirring, until pH ~ 5 - 6 was reached. Massively precipitating crystals were filtered off, washed with 50 ml cold water and dried with P_2O_5. Chemical purity of **8** was checked by TLC chromatography, R_f = 0.66 (IPA : NH_4OH conc. : water = 7 : 1 : 2).

Intermediate **8** (4.4 g; 28 mmol) was dissolved in 100 ml of saturated $NaHCO_3$, ice-cooled and Fmoc-*O*su (9.4 g; 28 mmol) in 100 ml dioxane was added dropwise. The reaction mixture was stirred for 1 hour at 0 °C and then overnight at rt. Then 200 ml of water was added, the flask was cooled again

in an ice bath and 1M HCl was added until pH ~ 1 was reached. The reaction mixture was extracted 3 x with 100 ml ethyl acetate; the combined organic phases were washed with 100 ml water, 100 ml brine, dried with Na_2SO_4 and concentrated *in vacuo* to give a crude oily material, which was subjected to flash chromatography on silica, using a linear gradient of 1 % AcOH/ethyl acetate in toluene. The product (10 g, light yellow oil) was dissolved during heating (60 °C) in a minimal amount of toluene, and the flask was placed at -20 °C overnight. Next, 50 ml of hexane was added to the gelatinous material, the mixture was sonicated for 10 minutes in an ice-cooled bath and then again placed at -20 °C overnight. The resulting crystals were filtered off, washed with 50 ml of chilled hexane and dried under deep vacuum. Yield 8.8 g (58 %) for 3 steps. Colorless solid, m.p. 140 - 142 °C. R_f = 0.73 (DCM : MeOH : NH_4OH conc. = 75 : 22 : 3). $[\alpha]\frac{20}{D}$ = -93.9 (c = 2 ; DMF); ^{1}H NMR (600 MHz, DMSO; T = 25 °C): 1.92 s (3H, CH_3−), 2.88 dd (1H, *J* = 13.3 and 10.1 Hz) and 3.16 dd (1H, *J* = 13.3 and 4.3 Hz) (−CH_2−S), 4.22 ddd (1H, *J* = 10.1, 8.2 and 4.3 Hz, >CH−N),4.24 t (1H, *J* = 7.1 Hz, >CH−), 4.32 d (2H, *J* = 7.1 Hz, (−CH_2−O), 7.33 td (2H, *J* = 7.5 and 1.0 Hz, 2x ArH (Fmoc)), 7.42 td (2H, *J* = 7.5 and 1.0 Hz, 2x ArH (Fmoc)), 7.73 dt (2H, *J* = 7.5 and 1.0 Hz, 2x ArH (Fmoc)), 7.78 d (1H, *J* = 8.2 Hz, >NH), 7.90 dt (2H, *J* = 7.5 and 1.0 Hz, 2x ArH (Fmoc)), 13.02 br (1H, COOH).^{13}C NMR (150.9 MHz, DMSO; T = 25 °C): 47.6 (CH_3−), 36.37 (−CH_2−S), 46.76 (>CH−(Fmoc)), 53.50 (>CH−N), 65.93 (−CH_2−O (Fmoc)), 66.58 (≡C−S), 91.14 (≡C−), 120.31 (2x =CH− (Fmoc)), 125.41 (2x =CH−(Fmoc)), 127.27 (2x =CH−(Fmoc)), 127.83 (2x CH−(Fmoc)), 140.91 (2x =C< (Fmoc)), 143.94 (=C< (Fmoc)), 143.95 (=C<(Fmoc)), 156.14 (O−CO−N), 172.00 (−COOH). IR (KBr, ν_{max} cm^{-1}) 2120 vw (C≡C); 1710 vs (C=O) acid, 1685 vs (C=O) carbamate; 1533 s, 1525 s (amide II); 3316 s (NH); 3065 m, 3043 m, 1611 w, 1579 w, 1478 m, 1450 s, 1104 w, 1034 w, 757 m, 739 s, 622 m (fluorene). HRMS (ESI) calculated for $C_{21}H_{18}O_4NS$ $[M-H]^-$ 380.09620; found: 380.09615.

(*R*)-2-amino-3-(prop-2-yn-1-ylthio)propanoic acid **10**

(L-Cys(Prp)-OH)

The compound was prepared according to the earlier described Method 2 (Ref. [2]) by the reaction of L-cysteine (5 g; 41.3 mmol) and 80 % propargyl bromide (6.9 ml; 62 mmol). Yield 3.6 g (56 %).

Beige solid, m. p. > 160 °C (decay). R_f = 0.58 (IPA : NH_4OH conc. : water = 7 : 1 : 2). $[\alpha]_D^{20}$ = -7.4 (c = 2 ; water); ^1H NMR (600 MHz, D_2O; T = 25 °C): 3.03 dd (1H, *J* = 14.0 and 7.3 Hz) and 3.14 dd (1H, *J* = 14.0 and 4.7 Hz) (−CH_2−S), 3.38 s (2H, (−CH_2−S), 3.69 dd (1H, *J* = 7.3 and 4.7 Hz, >CH−N). ^{13}C NMR (150.9 MHz, D_2O; T = 25 °C): 21.54 (−CH_2−S), 37.60 (−CH_2−S), 57.06 (>CH−N). 74.82 (triplet, ^1J(C,D) = 38.6 Hz, ≡CD), 82.81 (triplet, ^2J(C,D) = 7.5 Hz, ≡C−), 180.61 (COOH). Acetylenic hydrogen is exchanged in D_2O for deuterium. Therefore, its signal is not observed in ^1H NMR spectrum and the presence of deuterium is manifested by the splitting of acetylene carbon signals into triplets. IR (KBr, ν_{max} cm^{-1}) 3279 s (C≡C−H); 2115 w (C≡C); 1619 vs, 1506 vs (NH_3^+), 1589 vs, 1411 s (CO_2^-). HRMS (ESI) calculated for $C_6H_8O_2NS$ $[M-H]^-$ 158.02812; found: 158.02805.

(*R*)-2-{[((Fluorenyl-9-yl)methoxy)carbonyl]amino}-3-(prop-2-yn-1-ylthio)propanoic acid **11** (Fmoc-L-Cys(Prp)-OH)

Compound **11** was prepared using the slightly modified protocol published earlier [3] (including work-up and isolation previously employed for **9)** from acid **10** (3.8 g; 23.9 mmol) and Fmoc-*O*Su (8.1 g ; 23.9 mmol). Yield 8.1 g (89 %).

Alternatively, Fmoc-L-Cys-OH [4] (5.6 g; 16.4 mmol) and 80 % propargyl bromide (2.2 ml; 19.7 mmol) were dissolved in 100 ml of argon-bubbled ethyl acetate and the deaerated aqueous solution (100 ml) of $NaHCO_3$ (5.5 g; 65.6 mmol) and TBAB (0.52 g; 1.6 mmol) were added in one step. The reaction mixture was vigorously stirred in argon atmosphere at rt for 4 days. Afterwards, 1 M HCl was slowly added dropwise until pH ~ 1 was reached. The organic layer was separated and the aqueous phase was extracted 2 x with 100 ml ethyl acetate. The combined organic phases were washed with 100 ml water, 2 x 100 ml brine, dried on sodium sulfate and evaporated under reduced pressure to afford 8.7 g of oily residue. Flash chromatography and crystallization of the product were performed by the same method as for compound **9**. Yield 4.8 g (77 %). Colorless solid, m. p. 76 - 78 °C. R_f = 0.65 (DCM : MeOH : NH_4OH conc. = 75 : 22 : 3). $[\alpha]_D^{20}$ = -52.5 (c = 2 ; DMF); ^1H NMR (600 MHz, DMSO; T = 25 °C): 2.84 dd (1H, *J* = 13.6 and 9.6 Hz) and 3.10 dd (1H, *J* = 13.6 and 4.6 Hz) (−CH_2−S), 3.18 t (1H, *J* = 2.6 Hz, ≡CH), 3.36 dd (1H, *J* = 16.8 and 2.6 Hz) and 3.41 dd (1H, *J* = 16.8 and 2.6 Hz) (−CH_2−S), 4.18 ddd

(1H, *J* = 9.6, 8.4 and 4.6 Hz, >CH−N), 4.23 dd (1H, *J* = 7.4 and 6.7 Hz, >CH−), 4.28 dd (1H, *J* = 10.5 and 6.7 Hz) and 4.31 dd (1H, *J* = 10.5 and 7.4 Hz) (−CH_2−O), 7.33 td (2H, *J* = 7.5 and 1.0 Hz, 2x ArH (Fmoc)), 7.42 td (2H, *J* = 7.5 and 1.0 Hz, 2x ArH (Fmoc)), 7.73 dt (2H, *J* = 7.5 and 1.0 Hz, 2x ArH (Fmoc)), 7.74 d (1H, *J* = 8.4 Hz, >NH), 7.89 dt (2H, *J* = 7.5 and 1.0 Hz, 2x ArH (Fmoc)), 12.90 br (1H, COOH). ^{13}C NMR (150.9 MHz, DMSO; T = 25 °C): 18.86 (−CH_2−S), 32.66 (−CH_2−S), 46.80 (>CH−(Fmoc)), 53.66 (>CH−N), 65.92 (−CH_2−O (Fmoc)), 74.04 (≡CH), 80.38 (≡C−), 120.29 (2x =CH−(Fmoc)), 125.45 (=CH− (Fmoc)), 125.49 (=CH− (Fmoc)), 127.27 (2x =CH−(Fmoc)), 127.82 (2x CH− (Fmoc)), 140.89 (2x =C< (Fmoc)), 143.97 (2x =C< (Fmoc)), 156.17 (O−CO−N), 172.41 (−COOH). IR (KBr, ν_{max} cm^{-1}) 3294 s (C≡C−H); 2117 vw (C≡C); 1704 vs (C=O) acid; 1685 s (C=O) carbamate; 1536 s (amide II); 3320 s (NH); 2961 m, 2945 m (CH_2); 3064 m, 3040 m, 1610 w, 1579 w, 1478 m, 1451 m, 1104 m, 1035 m, 759 s, 739 s, 621 m (fluorene). HRMS (ESI) calculated for $C_{21}H_{18}O_4NS$ $[M\text{-}H]^-$ 380.09620; found: 380.09590.

tert-Butyl 2-(*S*)-(*tert*-butoxycarbonylamino)-3-hydroxypropanoate **12**

O-*tert*-Butyl-*N*,*N´*-diisopropyl isourea (9.7 g; 48.7 mmol) in 250 ml DCM was added to a stirred cloudy solution of Boc-L-Ser-OH (10 g; 48.7 mmol) and the reaction mixture was allowed to react at rt overnight. Then, 4 portions of isourea (1.9 g; 9.7 mmol) were added in 12-hour periods, until TLC analysis revealed the almost complete disappearance of the starting compound. The volatile material was evaporated under reduced pressure, the semi-solid residue was dissolved in 100 ml ethyl acetate and placed at -20 °C. After several hours, the precipitated crystals (urea) were filtered off and washed with chilled ethyl acetate (100 ml). The filtrate phase was evaporated *in vacuo* to afford a crude yellow oil (14 g), which was subjected to flash chromatography on silica using a linear gradient ethyl acetate in toluene. Yield 9.2 g (72 %). Clear viscous oil. The physical-chemical characteristics were in a full agreement with those published earlier [5].

tert-Butyl 2-(*S*)-(*tert*-butoxycarbonylamino)-3-bromopropanoate **13**

Compound **13** was prepared by the reaction of **12** (9.2 g; 35.2 mmol) with PPh_3 (10.2 g; 38.7 mmol) and CBr_4 (12.8 g; 38.7 mmol) as previously described by Pícha et al. [5]. Yield 7.5 g (66 %).

tert-Butyl 2-(*R*)-(*tert*-butoxycarbonylamino)-3-[(2-hydroxyethyl)sulfanyl]propanoate **14**

The slurry of **13** (7.5 g; 23.1 mmol), 2-mercaptoethanol (1.8 g; 23.1 mmol) and K_2CO_3 (3.2 g; 23.1 mmol) were stirred overnight in 150 ml of acetonitrile in argon atmosphere. The solid material was filtered off on Celite® and the filter pad was then washed with 200 ml ethyl acetate. The filtrate was evaporated under reduced pressure to give 7 g of colorless oil, which was subjected to flash chromatography on silica using a linear gradient of ethyl acetate in toluene. Yield 4.5 g (61 %).

Alternatively, **18** (9.1 g ; 34.3 mmol) and *O*-*tert*-butyl-*N,N´*-diisopropyl isourea (6.9 g ; 34.3 mmol) in 100 ml of DCM were allowed to react at rt overnight. Then, 5 portions of isourea (1.4 g; 6.9 mmol) were added in 12-hour periods, until TLC analysis (DCM : MeOH : NH_4OH conc. = 75 : 22 : 3) revealed almost complete consumption of the starting compound **18**. The volatile material was evaporated under reduced pressure, the residue was dissolved in 100 ml ethyl acetate and placed at -20 °C. After several hours, the precipitated DIU was filtered off and washed with 100 ml of chilled ethyl acetate. The filtrate was evaporated *in vacuo* to afford a crude yellow oil (6.9 g), which was subjected to flash chromatography on silica using a linear gradient of ethyl acetate in toluene. Yield 6.1 g (55 %). R_f = 0.48 (toluene : ethyl acetate 50 : 50). $[\alpha]_D^{20}$ = + 6.5 (c = 2 ; $CHCl_3$); ^1H NMR (600 MHz, $CDCl_3$; T = 25 °C): 1.44 s (9H, *t*-Bu), 1.47 s (9H, *t*-Bu), 2.76 m (2H, −CH_2−S), 2.90 dd (1H, *J* = 13.8 and 5.8 Hz) and 2.96 dd (1H, *J* = 13.8 and 5.0 Hz) (−CH_2−S), 3.73 m (2H, −CH_2−O), 4.41 m (1H, >CH−N), 5.37 br (>NH).^{13}C NMR (150.9 MHz, $CDCl_3$; T = 25 °C): 27.97 (3x CH_3 (*t*-Bu)), 28.29 (3x CH_3 (*t*-Bu)), 35.06 (−CH_2−S), 36.46 (−CH_2−S), 54.18 (>CH−N), 60.60 (−CH_2−O), 80.08 (>C< (*t*-Bu)), 82.76 (>C< (*t*-Bu)), 155.38 (O−CO−N), 169.92 (O−CO−). IR ($CHCl_3$, ν_{max} cm^{-1}) 3620 vw (OH); 3433 w (NH); 1730 vs (C=O) ester; 1707 vs (C=O) carbamate; 1497 s (ade II); 2982 m, 2878 w, 1394 m, 1370 s (CH_3); 2933 w, 1456 w (CH_2); 1154 vs (($CH_3)_3$). HRMS (ESI) calculated for $C_{14}H_{27}O_5NNaS$ $[M+Na]^+$ 344.15021; found: 344.15003.

tert-Butyl 2-(*R*)-(*tert*-butoxycarbonylamino)-3-[(2-bromoethyl)sulfanyl]propanoate **15**

Ester **14** (7.8 g; 24.3 mmol) and CBr_4 (8.9 g ; 26.7 mmol) were dissolved in 100 ml of DCM. The flask was ice-cooled and PPh_3 (7 g; 26.7 mmol) in 50 ml of DCM was slowly added dropwise during

stirring. Stirring was continued for 1 hour at 0 °C and then at rt overnight. The volatile material was removed on the evaporator under reduced pressure and the resulting yellow oil was dissolved (sonication) in a mixture of diethyl ether (500 ml) and petroleum ether (2 000 ml). The solution was placed at -20 °C overnight. The precipitate of triphenylphosphine oxide was filtered off and washed with a mixture of 500 ml chilled diethyl ether and petroleum ether (1 : 4). The filtrate was evaporated under reduced pressure to give 11.5 g of bright yellow oil, which was subjected to flash chromatography on silica using a linear gradient ethyl acetate in toluene. Yield 6.3 g (68 %). Colorless oil. R_f = 0.60 (toluene : ethyl acetate 90 : 10). $[\alpha]_D^{20}$ = + 1.9 (c = 2 ; $CHCl_3$). ^{1}H NMR (600 MHz, $CDCl_3$; T = 25 °C): 1.44 s (9H, *t*-Bu), 1.47 s (9H, *t*-Bu), 2.96 m (2H, −CH_2−S), 2.98 dd (1H, *J* = 13.8 and 5.0 Hz) and 3.03 dd (1H, *J* = 13.8 and 5.0 Hz) (−CH_2−S), 3.46 m (2H, −CH_2−Br), 4.40 dt (1H, *J* = 7.0 and 5.0 Hz, −CH−N), 5.35 d (1H, *J* = 7.0 Hz, >NH). ^{13}C NMR (150.9 MHz, $CDCl_3$; T = 25 °C): 27.96 (3x CH_3 (*t*-Bu)), 28.29 (3x CH_3 (*t*-Bu)), 30.11 (−CH_2−Br), 34.71 (−CH_2−S), 34.81 (−CH_2−S), 54.08 (>CH−N), 80.05 (>C< (*t*-Bu)), 82.85 (>C< (*t*-Bu)), 155.04 (O−CO−N), 169.57 (O−CO−). IR ($CHCl_3$, ν_{max} cm^{-1}) 3431 w (NH); 1730 vs (C=O) ester; 1708 vs (C=O) carbamate; 1497 s (amide II); 2982 m, 2872 w, 1394 m, 1370 s (CH_3); 2934 w, 1456 m (CH_2); 1154 vs ($(CH_3)_3$); 616 w (C-Br). HRMS (ESI) calculated for $C_{14}H_{26}O_4BrNNaS$ $[M+Na]^+$ 406.06581; found: 406.06576.

tert -Butyl 2-(*R*)-(*tert*-butoxycarbonylamino)-3-[(2-azidoethyl)sulfanyl]propanoate **16**

Ester **14** (6.1 g ; 19 mmol) was dissolved in 100 ml of DCM with TEA (4 ml; 28.5 mmol). The reaction mixture was ice-cooled and MsCl (1.6 ml; 20.9 mmol) was added dropwise. After 1 hour of stirring at 0 °C, TLC analysis (ethyl acetate : toluene 1 : 1) revealed complete consumption of the starting compound **14**. Excess of TEA was removed by addition of 10 % citric acid, the organic phase was separated, washed with 50 ml water and 2 x 50 ml brine and dried on Na_2SO_4. The filtrate was evaporated under reduced pressure to give 7.7 g of clear oil. The crude mesylated-intermediate was dissolved in 70 ml of anhydrous DMSO with NaN_3 (5 g; 76 mmol) and heated at 70 °C overnight. After cooling, 150 ml water was added, and the solution was extracted with 4 x 100 ml ethyl acetate. The combined organic phases were washed successively with 1 x 100 ml water and 2 x 100 ml brine and dried over Na_2SO_4.

The filtrate was evaporated under reduced pressure to give 8 g of yellow oil, which was subjected to flash chromatography on silica using a linear gradient of ethyl acetate in toluene. Yield 5.3 g (80 %, for 2 steps).

Alternatively, **15** (5.8 g; 15.1 mmol) and NaN_3 (2 g; 70.2 mmol) were heated at 70 °C in 40 ml of anhydrous DMSO overnight. After cooling, 100 ml water was added and the solution was extracted with 3 x 100 ml ethyl acetate. The combined organic phases were washed with 50 ml water and 2 x 50 ml brine and dried on Na_2SO_4. The filtrate was evaporated under reduced pressure to give 5.5 g of yellow oil, which was subjected to flash chromatography on silica using a linear gradient of ethyl acetate in toluene. Yield 4.7 g (90 %). Colorless oil. R_f = 0.57 (toluene : ethyl acetate 90 : 10). $[\alpha]_D^{20}$ = + 4.0 (c = 2; $CHCl_3$). ^{1}H NMR (600 MHz, $CDCl_3$; T = 25 °C): 1.44 s (9H, *t*-Bu), 1.48 s (9H, *t*-Bu), 2.75 t (2H, *J* = 6.9 Hz, −CH_2−S), 2.98 dd (1H, *J* = 13.8 and 5.0 Hz) and 3.03 dd (1H, *J* = 13.8 and 5.0 Hz) (−CH_2−S), 3.45 t (2H, *J* = 6.9 Hz, −CH_2−N_3), 4.40 dt (1H, *J* = 7.2 and 5.0 Hz, >CH−N), 5.36 br d (1H, *J* = 6.9 Hz, >NH). ^{13}C NMR (150.9 MHz, $CDCl_3$; T = 25 °C): 27.96 (3x CH_3 (*t*-Bu)), 28.29 (3x CH_3 (*t*-Bu)), 32.10 (−CH_2−S), 34.88 (−CH_2−S), 51.03 (−CH_2−N_3), 54.04 (>CH−N), 80.00 (>C< (*t*-Bu)), 82.77 (>C< (*t*-Bu)), 155.11 (O−CO−N), 169.82 (O−CO−). IR ($CHCl_3$, ν_{max} cm^{-1}) 3431 w (NH); 2104 vs (N_3); 1730 vs (C=O) ester; 1708 vs (C=O) carbamate; 1497 s (amide II); 2983 m, 2871 w, 1394 m, 1370 s (CH_3); 2933 w, 1456 m (CH_2); 1154 vs ($(CH_3)_3$). HRMS (ESI) calculated for $C_{14}H_{26}O_4N_4NaS$ $[M+Na]^+$ 369.15670; found: 369.15672.

2-(*R*)-(*tert*-Butoxycarbonylamino)-3-[(2-hydroxyethyl)sulfanyl]propanoic acid **18**

L-Cysteine (9.7 g; 80 mmol) in 250 ml of deaerated methanol was placed in a 500 ml round-bottom flask, equipped with a magnetic rod, and surrounded by an ice-bath. Stirring was started under the protective atmosphere of argon, and small portions of sodium (3.9 g; 168 mmol) were added carefully during a 1 h period. When the slurry became clear (approx. after 0.5 hour), 2-bromoethanol (10 g; 80 mmol) was added dropwise and the reaction mixture was allowed to react at rt overnight. Methanol was evaporated under reduced pressure, the residue was dissolved in 50 ml water and the solution was applied on a column of Dowex in H^+ form (160 g; 5.2 ± 0.3 meq/g), which was then washed

with 750 ml of a mixture of methanol-water (1 : 9). The product was liberated from the ion-exchange resin by washing with 1.5 l of mixture of conc. NH_4OH : MeOH : water (1 : 1 : 3). The collected filtrate was evaporated under reduced pressure on an evaporator; the resulting slurry of crystals was co-evaporated with 2 x 500 ml of toluene, and dried under deep vacuum overnight. The solid material was dissolved at 80 °C in 30 ml water and then 120 ml of 96 % ethanol was added. The clear solution was allowed to stand overnight at rt. The precipitate was filtered off and the crystals (6.4 g) were washed with 50 ml of chilled ethanol. The mother liquor was concentrated on the evaporator, and crystallization from water (10 ml) and ethanol (80 ml) produced the second portion of crystals (4.8 g). TLC analysis (IPA : conc. NH_4OH : water = 7 : 1 : 2) revealed identical profiles for both samples: major spot (R_f = 0.50) of 2-(*R*)-3-[(2-hydroxyethyl)sulfanyl]propanoic acid **17** and minor spot (R_f = 0.32) of unknown impurity. The material was used for further reaction without additional purification.

Compound **17** (5 g; 30.3 mmol) and Na_2CO_3 (6.4 g ; 60.6 mmol) were dissolved in 150 ml water and stirred. Then Boc anhydride (6.6 g ; 30.3 mmol) in 100 ml of dioxane was added dropwise at 0 °C during a period of 0.5 hour. The cooling bath was removed, and the reaction mixture was allowed to react overnight. Thereafter, 500 ml water was added, the solution was saturated with sodium chloride and acidified to pH ~ 3 with 10 % citric acid while being efficiently cooled with ice. The reaction mixture was extracted 3 x with 150 ml of ethyl acetate; the combined organic phases were washed 3 x with 100 ml brine and dried on Na_2SO_4, filtered and the solvent was removed under reduced pressure. The oily residue was subjected to flash chromatography on silica using linear gradient of ethyl acetate : methanol : acetone (4 : 3 : 1) in ethyl acetate. Yield 6.1 g (76 %). Colorless and viscous oil. R_f = 0.24 (DCM : MeOH : NH_4OH conc. = 75 : 22 : 3). $[\alpha]_D^{20}$ = + 17.6 (c = 2 ; $CHCl_3$). ^1H NMR (600 MHz, $CDCl_3$; T = 25 °C): 1.44 s (9H, *t*-Bu), 2.77 m (2H, $-CH_2-S$), 2.99 dd (1H, J = 13.8 and 6.0 Hz) and 3.04 dd (1H, J = 13.8 and 5.0 Hz) ($-CH_2-S$), 3.76 t (2H, J = 5.9 Hz, $-CH_2-O$), 4.56 m (1H, >CH−), 5.60 br (1H, >NH). ^{13}C NMR (150.9 MHz, $CDCl_3$; T = 25 °C): 28.27 (3x CH_3 (*t*-Bu)), 34.72 ($-CH_2-S$), 35.65 ($-CH_2-S$), 53.37 (>CH−N), 60.96 ($-CH_2-O$), 80.67 (>C< (*t*-Bu)), 155.75 (O−CO−N), 173.91 (−COOH). IR ($CHCl_3$, ν_{max} cm^{-1}) 3617 vw (OH); 3433 w (NH); 1725 vs (C=O) acid; 1710 vs (C=O) carbamate; 1502 s (amide II); 2983 m, 2882 w, 1394 m, 1370 s (CH_3); 2933 w, 1455 m (CH_2); 1163 vs

(($CH_3)_3$); 1058 s (C-OH). HRMS (ESI) calculated for $C_{10}H_{18}O_5NS$ $[M-H]^-$ 264.09112; found: 264.09107.

2-(*R*)-(9H-Fluoren-9-ylmethoxycarbonylamino)-3-[(2-hydroxyethyl)sulfanyl]propanoic acid **19**

Sodium (1.9 g; 84 mmol) was added under argon atmosphere in several portions to the ice-cooled and stirred slurry of L-cysteine (4.8 g; 40 mmol) in 150 ml of deaerated methanol. After 30 min when the suspension had changed to a clear solution, 2-bromoethanol (5 g; 40 mmol) was added in one portion and the reaction was allowed to react for 3 h at rt. Next, acidity was adjusted to pH ~ 3 with 5 % HCl. The volatile material was evaporated under reduced pressure, the residue was dissolved in 150 ml water and the pH of the reaction mixture was adjusted to ~ 8 by with solid $NaHCO_3$. The flask was ice-cooled and Fmoc-*O*Su (13.5 g; 40 mmol) in 150 ml dioxane was added dropwise. The reaction mixture was allowed to react overnight and then acidified with 1M HCl to pH ~ 1. The white precipitate was solubilized in 5 x 100 ml of ethyl acetate and the combined organic layers were washed with 2 x 100 ml of brine and dried on sodium sulfate. The filtrate was evaporated to give a solid material, which was recrystallized twice from a mixture ethyl acetate-hexane. Yield 11.1 g (73 % for 2 steps). Colorless solid, m.p. 116 – 117 °C. R_f = 0.50 (DCM : MeOH : NH_4OH conc. = 75 : 22 : 3). $[\alpha]\frac{20}{D}$ = - 35.0 (c = 2 ; DMF). ^1H NMR (600 MHz, $CDCl_3$; T = 25 °C): 2.60 m (2H, −CH_2−S), 2.77 dd (1H, *J* = 13.6 and 9.3 Hz) and 2.96 dd (1H, *J* = 13.6 and 4.4 Hz) (−CH_2−S), 3.54 t (2H, *J* = 6.8 Hz, −CH_2−O), 4.13 ddd (1H, *J* = 9.3, 8.5 and 4.4 Hz, >CH−N), 4.23 dd (1H, *J* = 7.5 and 6.6 Hz, >CH−), 4.27 dd (1H, *J* = 10.4 and 6.6 Hz) and 4.30 dd (1H, *J* = 10.6 and 7.5 Hz) (−CH_2−O), 7.33 td (2H, *J* = 7.5 and 1.0 Hz, 2x ArH (Fmoc)), 7.42 td (2H, *J* = 7.5 and 1.0 Hz, 2x ArH (Fmoc)), 7.73 dt (2H, *J* = 7.5 and 1.0 Hz, 2x ArH (Fmoc)), 7.89 dt (2H, *J* = 7.5 and 1.0 Hz, 2x ArH (Fmoc)), 7.74 d (1H, *J* = 8.5 Hz, >NH). ^{13}C NMR (150.9 MHz, $CDCl_3$; T = 25 °C): 33.30 (−CH_2−S), 34.51 (−CH_2−S), 46.84 (>CH− (Fmoc)), 54.46 (>CH−N), 61.04 (−CH_2−O), 65.99 (−CH_2−O (Fmoc)), 83.07 (>C< (*t*-Bu)), 120.34 (2x =CH− (Fmoc)), 125.53 (=CH− (Fmoc)), 125.55 (=CH− (Fmoc)), 127.33 (2x =CH− (Fmoc)), 127.89 (2x =CH− (Fmoc)), 140.94 (2x =C< (Fmoc)), 144.01 (2x =C< (Fmoc)), 158.28 (O−CO−N), 172.59 (−COOH). IR (KBr, ν_{max} cm^{-1}) 3407 s (OH); 3310 s (NH); 1718 vs (C=O) acid; 1690 vs (C=O) carbamate; 1531 s (amide II); 3065 m, 3039

m, 1611 w, 1578 w, 1478 m, 1464 m, 1451 m, 1220 s, 1194 s, 1104 m, 1035 s, 796 w, 758 s, 738 s (fluoren); 1047 s (C-OH). HRMS (ESI) calculated for $C_{20}H_{20}O_5NS$ $[M-H]^-$ 386.10677; found: 386.10650.

tert-Butyl 2-(*R*)-(9H-fluoren-9-ylmethoxycarbonylamino)-3-[(2-hydroxyethyl)sulfanyl]-propanoate **20**

Compound **19** (6.2 g; 16 mmol) and *O*-*tert*-butyl-*N,N´*-diisopropyl isourea (3.2 g; 16 mmol) in 100 ml THF were allowed to react at rt overnight. After 12 hours, an additional portion of isourea (3.2 g; 16 mmol) was added and the reaction stirred for another 12 hours. The solid material was filtered off; the filtrate was evaporated and the residue was subjected to flash chromatography on silica using linear gradient of ethyl acetate in toluene. Yield 5.5 g (77 %). Colorless and viscous oil. R_f = 0.50 (toluene : ethyl acetate 50 : 50). $[\alpha]_D^{20}$ ~ 0 (c = 2 ; $CHCl_3$). ^1H NMR (600 MHz, $CDCl_3$; T = 25 °C): 1.50 s (9H, *t*-Bu), 2.76 m (2H, −CH_2−S), 2.96 dd (1H, *J* = 13.8 and 5.6 Hz) and 3.01 dd (1H, *J* = 13.8 and 4.7 Hz) (−CH_2−S), 3.73 m (2H, −CH_2−O), 4.24 t (1H, *J* = 7.4 and 7.0 Hz, >CH−), 4.39 dd (1H, *J* = 10.6 and 7.0 Hz) and 4.43 dd (1H, *J* = 10.6 and 7.4 Hz) (−CH_2−O), 5.76 br d (1H, *J* = 7.4 Hz, >NH), 7.32 td (2H, *J* = 7.5 and 1.0 Hz, 2x ArH (Fmoc)), 7.40 td (2H, *J* = 7.5 and 1.0 Hz, 2x ArH (Fmoc)), 7.62 dt (2H, *J* = 7.5 and 1.0 Hz, 2x ArH (Fmoc)), 7.77 dt (2H, *J* = 7.5 and 1.0 Hz, 2x ArH (Fmoc)). ^{13}C NMR (150.9 MHz, $CDCl_3$; T = 25 °C): 27.96 (3x CH_3 (*t*-Bu)), 35.06 (−CH_2−S), 36.45 (−CH_2−S), 47.10 (>CH− (Fmoc)), 54.56 (>CH−N), 60.73 (−CH_2−O), 67.11 (−CH_2−O (Fmoc)), 83.07 (>C< (*t*-Bu)), 119.97 (=CH− (Fmoc)), 119.98 (=CH− (Fmoc)), 125.09 (2x =CH− (Fmoc)), 127.05 (2x =CH− (Fmoc)), 127.70 (2x =CH− (Fmoc)), 141.27 (=C< (Fmoc)), 141.30 (=C< (Fmoc)), 143.72 (=C< (Fmoc)), 143.81 (=C< (Fmoc)), 155.82 (O−CO−N), 169.62 (−CO−O). IR ($CHCl_3$, ν_{max} cm^{-1}) 3620 w (OH); 3427 w (NH); 1731 vs (C=O) ester; 1718 vs (C=O) carbamate; 1506 s (amide II); 3068 m, 1604 vw, 1580 vw, 1478 m, 1465 m, 1451 m, 1105 w, 1034 m (fluoren); 2983 m, 2878 w, 1395 m, 1371 s (CH_3); 1154 s ($(CH_3)_3$); 1058 s (C-OH). HRMS (ESI) calculated for $C_{24}H_{30}O_5NS$ $[M+H]^+$ 444.18392; found: 444.18374.

2-(*R*)-(9H-Fluoren-9-ylmethoxycarbonylamino)-3-[(2-azidoethyl)sulfanyl]propanoic acid **23**

Potassium hydroxide (4.1 g; 73.4 mmol) in 50 ml methanol was added dropwise under argon atmosphere to a stirred slurry of L-cysteine (4.4 g; 36.7 mmol) in 100 ml of deaerated methanol. After

10 minutes, the suspension turned into a completely clear solution. Then, the protected bromide **24** (8.2 g; 36.7 mmol) in 50 ml of THF was added in one portion and the reaction was allowed to react overnight at rt. The solid citric acid was slowly added until pH ~ 6 was reached and methanol was evaporated under reduced pressure. Thereafter, the resulting bulky precipitate was dissolved in 150 ml water; the pH value of the turbid solution was adjusted to 8 with $NaHCO_3$ and Fmoc-*O*Su (12.4 g; 36.7 mmol) in 150 ml of dioxane was added dropwise during stirring and ice-cooling. After 12 hours of stirring at rt, the reaction mixture was acidified to pH ~ 3 with 1 M citric acid. Next, 250 ml water was added, and the reaction mixture was extracted with 3 x 150 ml of ethyl acetate. The combined organic phases were washed with 1 x 150 ml water, 2 x 150 ml brine and dried on Na_2SO_4. The filtrate was evaporated under reduced pressure and the yellow oil was subjected to flash chromatography on silica using linear gradient of ethyl acetate in toluene to afford 12.2 g 2-(*R*)-(9H-fluoren-9-ylmethoxycarbonylamino)-3-[(2-*tert* butoxycarbonylaminoethyl)sulfanyl]propanoic acid **22**. HPLC analysis (see Figure S7) revealed satisfactory purity (92 % rel.) of **22**, which enabled a diazo-transfer reaction to be performed.

12.2 g of **22** was treated with an acidic cocktail, consisting of 40 ml of DCM, 60 ml of TFA and 3 ml of TIPS. When the dramatic release of gas slowed down, the reaction mixture was allowed to react for another 2 hours. Volatile materials were evaporated under reduced pressure and the resulting yellow oil was dissolved in a mixture of 200 ml methanol and 200 ml saturated $NaHCO_3$. Then, a solution of freshly prepared triflic azide in 100 ml of DCM (prepared according to previously published protocol [4] from 50.2 mmol of triflic anhydride and 251 mmol of NaN_3) was added dropwise and the reaction mixture was allowed to react overnight at rt. The pH was adjusted to ~ 6 by adding 10 % citric acid and volatile solvents (MeOH, DCM) were evaporated under reduced pressure. Water (200 ml) was added to the resulting dense slurry and the mixture was acidified with 1 M HCl. The reaction mixture was extracted with 3 x 150 ml of ethyl acetate, and the combined organic layers were washed with 150 ml water, 2 x 100 ml brine and dried on Na_2SO_4. Sodium sulfate was filtered off and the filtrate evaporated. The residues were purified by flash chromatography on silica gel using linear gradient of ethyl acetate in toluene. Trituration of light brown oil in a mixture toluene-hexane at -20 °C gave compound **23**. The sample was purified by HPLC (Method 2). Yield 7.5 g (50 % for 3 steps). Semisolid. R_f = 0.58 (ethyl

acetate : MeOH : acetone : water = 6 : 1 : 1 : 0.5). $[\alpha]_D^{20}$ = -28 (c = 2 ; DMF). ^{1}H NMR (600 MHz, DMSO; T = 25 °C): 2.75 t (2H, *J* = 6.7 Hz, −CH_2−S), 2.80 dd (1H, *J* = 13.7 and 9.6 Hz) and 3.00 dd (1H, *J* = 13.7 and 4.6 Hz) (−CH_2−S), 3.49 t (1H, *J* = 6.7 Hz, −CH_2−N_3), 4.15 ddd (1H, *J* = 9.6, 8.6 and 4.6 Hz, >CH−N), 4.24 t (1H, *J* = 7.0 Hz, >CH−), 4.30 d (2H, *J* = 7.0 Hz, CH_2−O), 7.33 td (2H, *J* = 7.5 and 1.0 Hz, 2x ArH (Fmoc)), 7.42 td (2H, *J* = 7.5 and 1.0 Hz, 2x ArH (Fmoc)), 7.73 dt (2H, *J* = 7.5 and 1.0 Hz, 2x ArH (Fmoc)), 7.76 d (1H, *J* = 8.6 Hz, NH), 7.89 dt (2H, *J* = 7.5 and 1.0 Hz, 2x ArH (Fmoc)), 13.90 br (1H, COOH). ^{13}C NMR (150.9 MHz, DMSO; T = 25 °C): 31.16 (−CH_2−S), 32.99 (−CH_2−S), 46.83 (>CH− (Fmoc)), 50.51 (−CH_2−N_3), 54.46 (>CH−N), 65.96 (−CH_2−O (Fmoc)), 120.32 (2x =CH− (Fmoc)), 125.47 and 125.49 (2x =CH− (Fmoc)), 127.28 (2x =CH− (Fmoc)), 127.86 (2x =CH− (Fmoc)), 140.93 and 140.94 (2x =C< (Fmoc)), 143.99 and 144.00 (2x =C< (Fmoc)), 156.25 (O−CO−N), 172.40 (COOH). IR (KBr, ν_{max} cm^{-1}) 3317 w (NH); 2105 s (N_3); 1716 vs (C=O) acid; 1701 vs (C=O) carbamate; 1535 s (amide II); 3068 w, 3040 w, 1612 w, 1579 vw, 1478 w, 1451 m, 1104 w, 1034 m, 759 m, 740 s (fluoren). HRMS (ESI) calculated for $C_{20}H_{19}O_4N_4S$ $[M-H]^-$ 411.11325; found: 411.11318.

2-(*tert* Butoxycarbonylamino)ethyl bromide **24**

N-Boc-ethanolamine (12 g; 77.5 mmol) and (28.3 g; 85.5 mmol) CBr_4 were dissolved in 250 ml of DCM. The flask was ice-cooled, stirred and PPh_3 (22.5 g; 85.5 mmmol) in 100 ml of DCM was slowly added dropwise. The reaction mixture was allowed to react for 1 hour at 0 °C and then at rt overnight. Volatile material was removed under reduced pressure and the resulting yellow oil was sonicated in a mixture of 500 ml of diethyl ether and petroleum ether (1 : 4), until the oil was completely solubilized. The solution was placed at -20 °C overnight. The resulting precipitate of triphenylphosphine oxide was filtered off and washed with a mixture of 500 ml of chilled mixture diethyl ether and petroleum ether (1 : 4). The filtrate was evaporated under reduced pressure to give 30 g of bright yellow oil, which was subjected to flash chromatography on silica using a linear gradient ethyl acetate in toluene. Yield 12 g (69 %). Bright yellowish oil. R_f = 0.44 (toluene : ethyl acetate 90 : 10). f^{1}H NMR (600 MHz, $CDCl_3$; T = 25 °C): 1.44 s (9H, *t*-Bu), 3.45 t (2H, *J* = 5.7 Hz, −CH_2−Br), 3.52 m (2H, −CH_2−N), 4.96 br (1H, >NH). ^{13}C NMR (150.9 MHz, $CDCl_3$; T = 25 °C): 28.32 (3x CH_3 (*t*-Bu)), 32.81 (−CH_2−Br), 42.33

(−CH_2−N), 79.81 (>C< (*t*-Bu)), 155.59 (O−CO−N). IR ($CHCl_3$, ν_{max} cm^{-1}) 3457 m (NH); 2981 s, 2872 w, 1393 m, 1368 m (CH_3); 2933 m (CH_2); 1711 vs (C=O) carbamate; 1505 vs (amide II); 1167 s ($(OCH_3)_3$). HRMS (EI) calculated for $C_7H_{15}O_2NBr$ $[M+H]^+$ 224.0286; found: 224.0282.

*Analytical chromatograms of compounds **7**, **9**, **11**, **19**, **22** and **23***

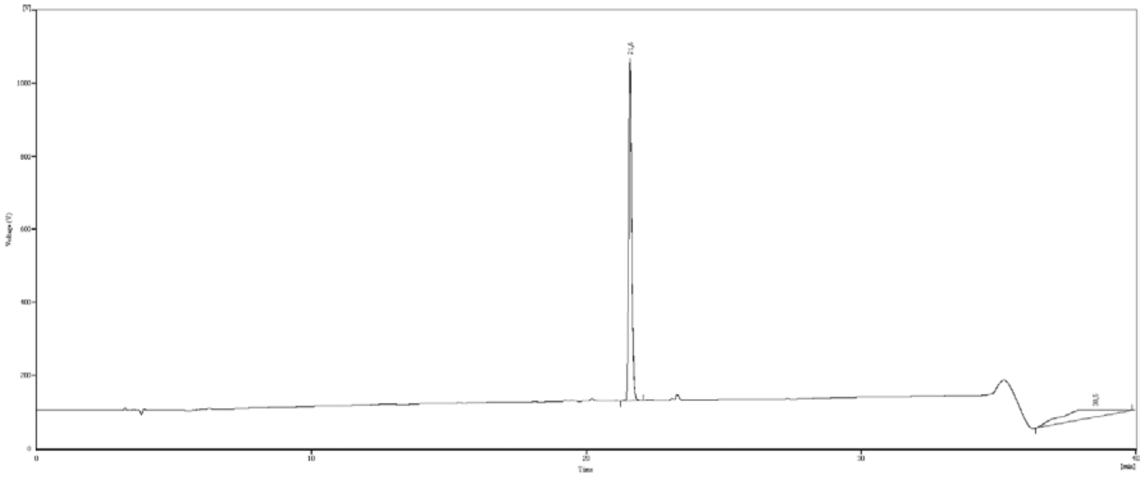

Figure S2. HPLC profile of purified compound **7** using a gradient from Method 1.

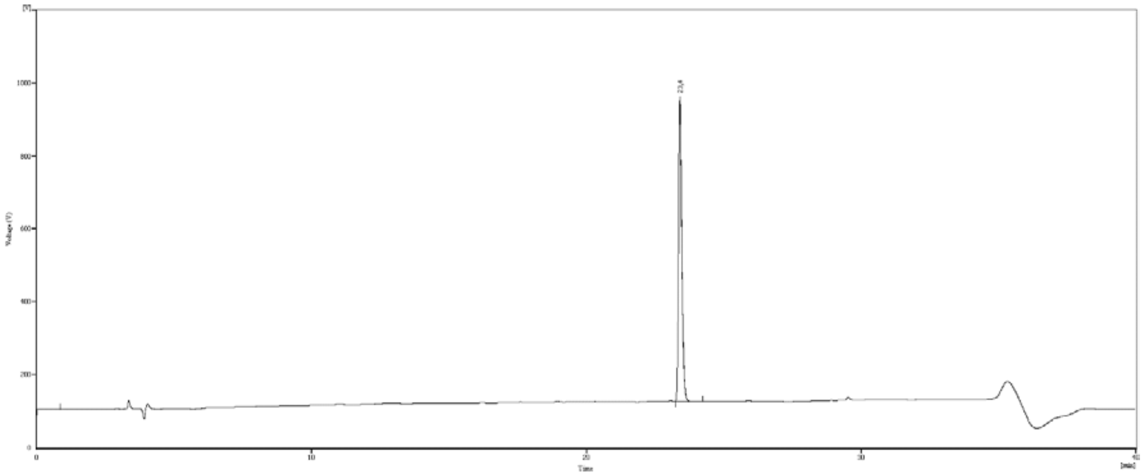

Figure S3. HPLC profile of purified compound **9** using a gradient from Method 1.

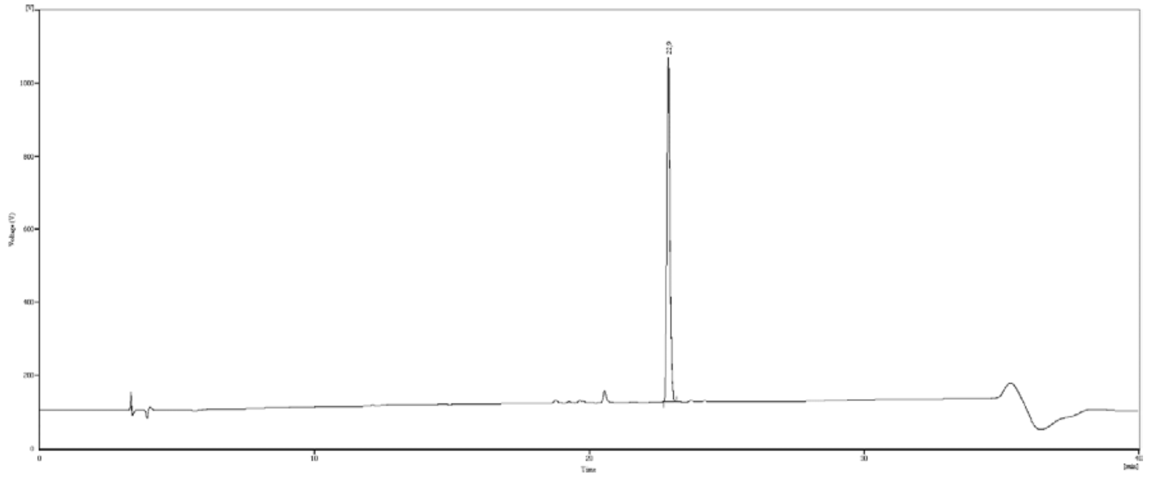

Figure S4. HPLC profile of purified compound **11** (prepared from compound **10**) using a gradient from Method 1.

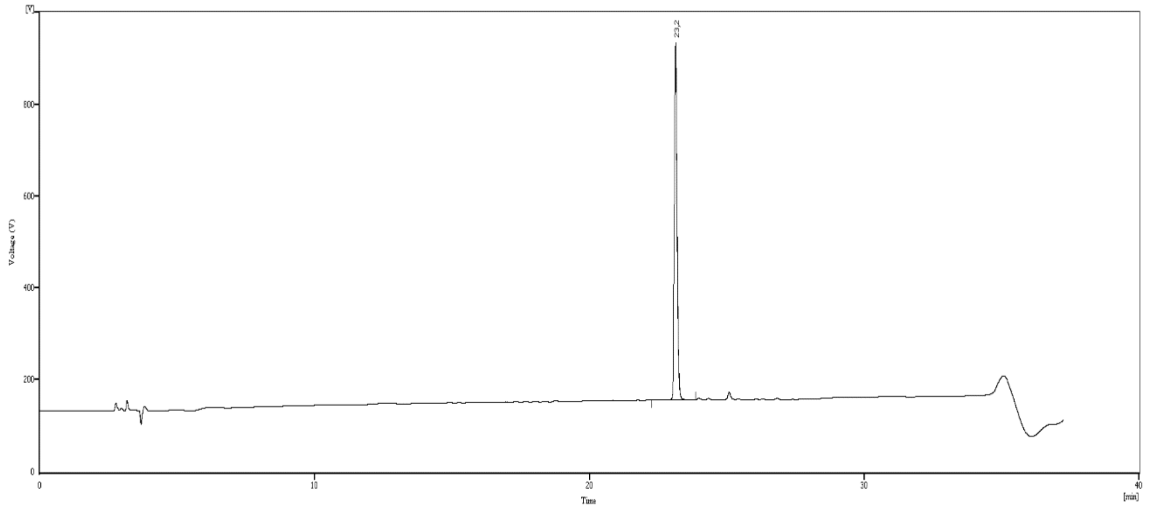

Figure S5. HPLC profile of purified compound **11** (prepared Fmoc-L-Cys-OH) using a gradient from Method 1.

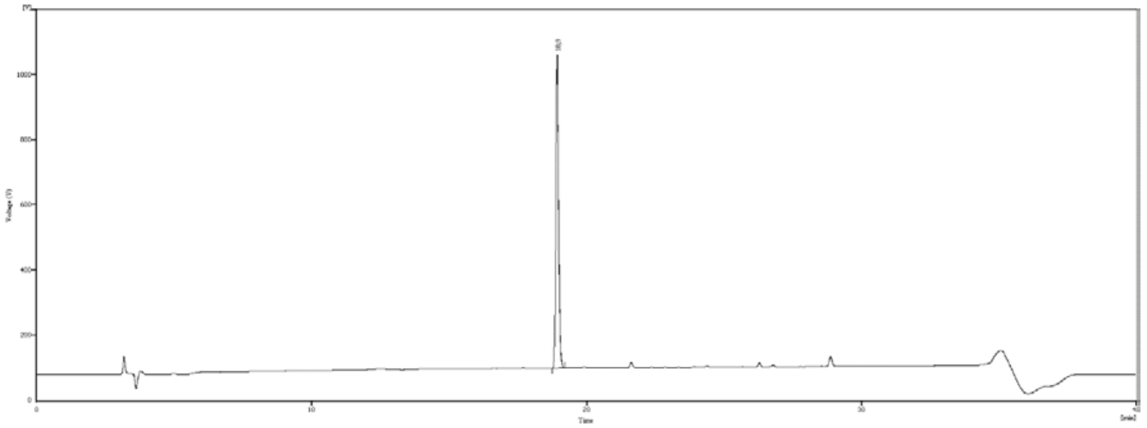

Figure S6. HPLC profile of purified compound **19** using a gradient from Method 1.

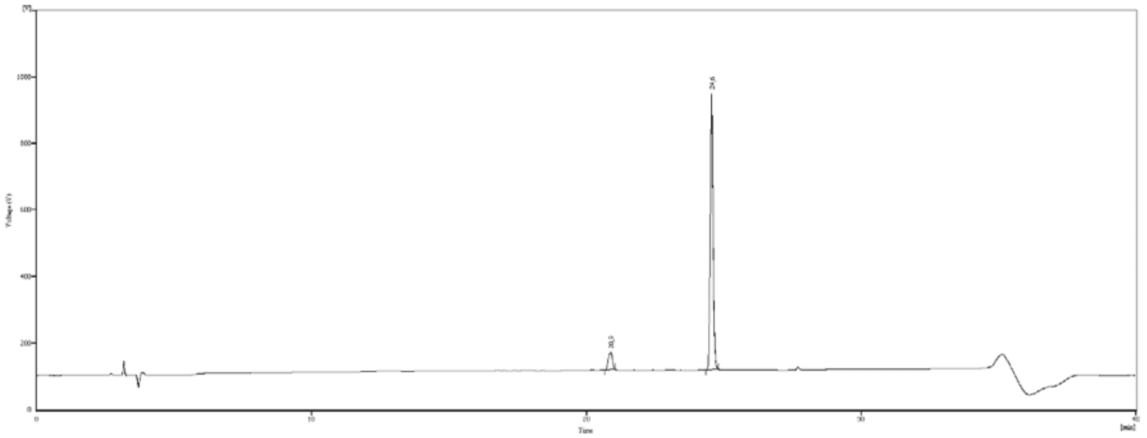

Figure S7. HPLC profile of crude compound **22** using a gradient from Method 1.

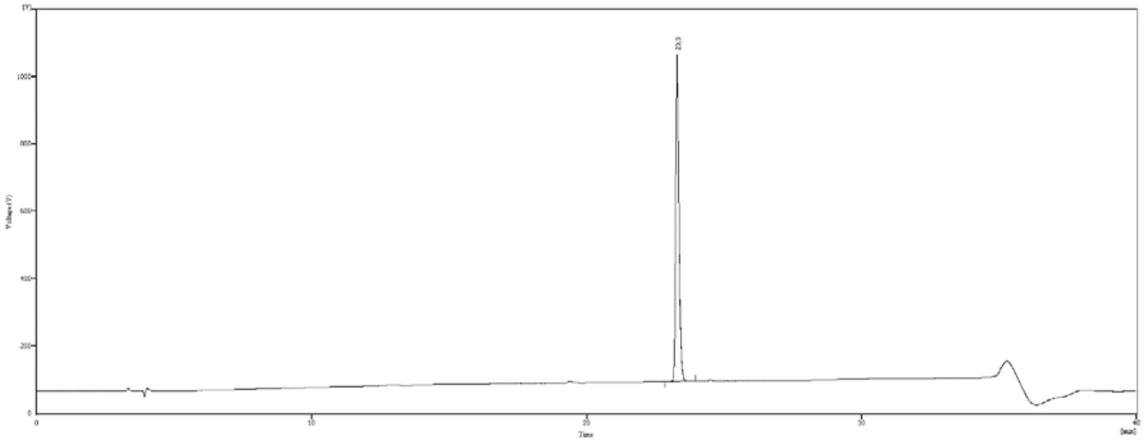

Figure S8. RP-HPLC profile of purified compound **23** using a gradient from Method 1.

Solid-phase peptide synthesis of peptides ***1-5***

Peptides were synthesized by solid-phase peptide synthesis (SPPS) on Rink Amide AM resin, using standard Fmoc protocol on the Spyder Mark IV Multiple Peptide Synthesizer (EP 17206537.7), developed in the Development Center of the Institute of Organic Chemistry and Biochemistry (http://dc.uochb.cz/index.php) (Figure S9).

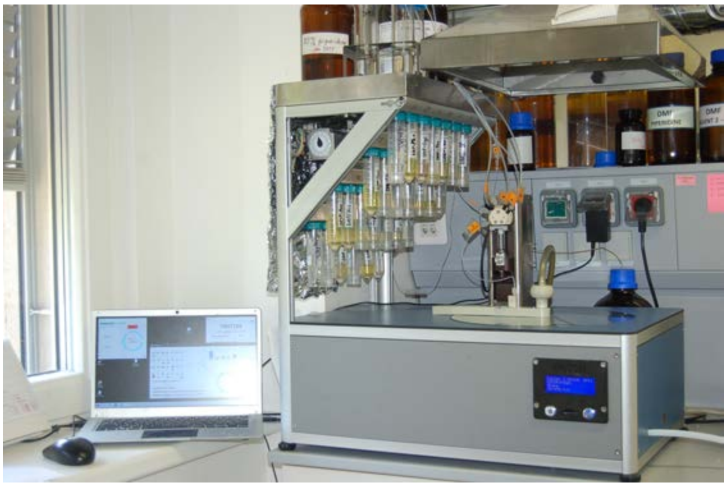

Figure S9. Spyder Mark IV Multiple Peptide Synthesizer (http://dc.uochb.cz/index.php).

Stock solutions of amino acids and reagents were used as follows: 0.4 M Fmoc-protected amino acids solubilized in 0.45 M HOBT in DMF, 20 % 4-methylpiperidine, 0.4 M HBTU in DMF, 1 M DIC in DMF and 1 M DIPEA in DMF.

The rink amide resin was placed in 12-ml syringes equipped with polypropylene frites and allowed to swell in DMF for 1 hour. Coupling reactions with natural amino acids were performed automatically in the Spyder Mark IV Synthesizer. Non-standard amino acids (**7**, **11**, **23**, Fmoc-azido-L-norvaline [5], Fmoc-L-propargylglycine and (*S*)-2-((((9H-fluoren-9-yl)methoxy)carbonyl)amino)-2-methylhept-6-enoic acid) were attached manually, as described earlier [6].

Generally, two couplings for 1h were done for each position of the peptides. The first coupling was done with 4 eq. of Fmoc-amino acid and 4.1 eq. of DIC. The second coupling was done with 4 eq. of Fmoc-amino acid, 3.8 eq. of HBTU and 7.6 eq. of DIPEA. The Fmoc group was cleaved with 20 %

4-methylpiperidine in DMF for 2 and 20 min. After the couplings and Fmoc group deprotection, the resin was washed 5 x 2 min with 2 ml of DMF.

Peptides were cleaved from the resin by treatment with an acidic cocktail (TFA : TIPS : H_2O = 95 : 2.5 : 2.5 v/v) for 2 hours. Crude peptides were precipitated with chilled ether, dried, dissolved in a mixture of CAN and water and lyophilized. Peptides were purified by a Waters HPLC system (Waters 600 with 2487 Dual λ Absorbance Detector), using a Nucleosil 100-7 C8 column (250 x 10 mm, 7 µm, Macherey-Nagel) at a flow rate of 4 ml/min and the following gradient: t = 0 min/10 % B, t = 30 min/100 % B, t = 31 min/10 % B. Solvent A is 0.1 % TFA in water and solvent B is 80 % ACN in A (v/v). Compounds were detected at 218 and 254 nm. The purity of the final peptides was checked by using HPLC on a Watrex HPLC system (Watrex DeltaChrom™ P200 binary Pump and Wufeng LC-100 UV Detector), using a Nucleosil 120-5 C8 column (250 x 4.6 mm, 5 µm, Macherey-Nagel) at a flow rate of 1 ml/min, with the same gradient and solvents as described for the preparative HPLC.

*Formation of disulfide bridge in peptide **1** (Scheme S1)*

Peptide **1** with Acm protection of cysteine thiol groups was dissolved at a concentration 10^{-3} M in 40 % AcOH. Iodine (25 eq.) in AcOH was added. The resulting solution was stirred at RT for 20 min and then 1 M ascorbic acid in water was added until the dark iodine color disappeared. Peptide **1** was purified as described above.

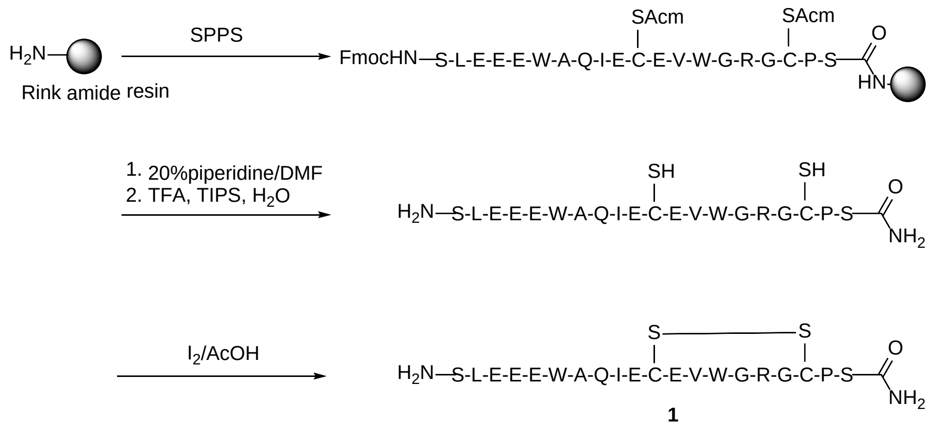

Scheme S1. Preparation peptide **1**.

*Staple formation by ring-closing olefin metathesis (RCM) in peptide **2** (Scheme S2)*

RCM was performed on the resin-bound peptide with the *N*-terminal amino acid protected with Fmoc. RCM was carried out using freshly prepared 6 mM Grubbs catalyst 1st generation (20 mol% regarding the resin substitution) in 1,2-dichloroethane (DCE). The resin was agitated under nitrogen at rt for 2 h protected from light. The reaction was repeated once more and a test cleavage with MS analysis was performed. If the RCM reaction was incomplete, it was repeated until completion. Thereafter, the Fmoc group was removed, and the peptide cleaved from resin and purified as described above. Synthetic scheme S10 shows the preparation of peptide **2**.

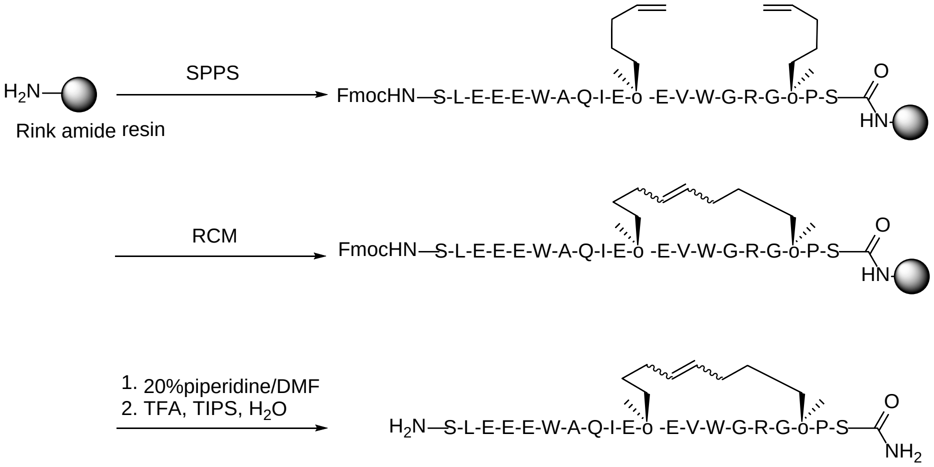

2

Scheme S2. Synthetic scheme of preparation peptide **2**. o denotes C^{α} atom of non-standard amino acids.

Cu(I)-catalyzed azide-alkyne cycloaddition (CuAAC, click reaction) of peptides ***3-5*** *(Schemes 3-5)*

CuAAC reactions were performed according to the protocol published previously [6].

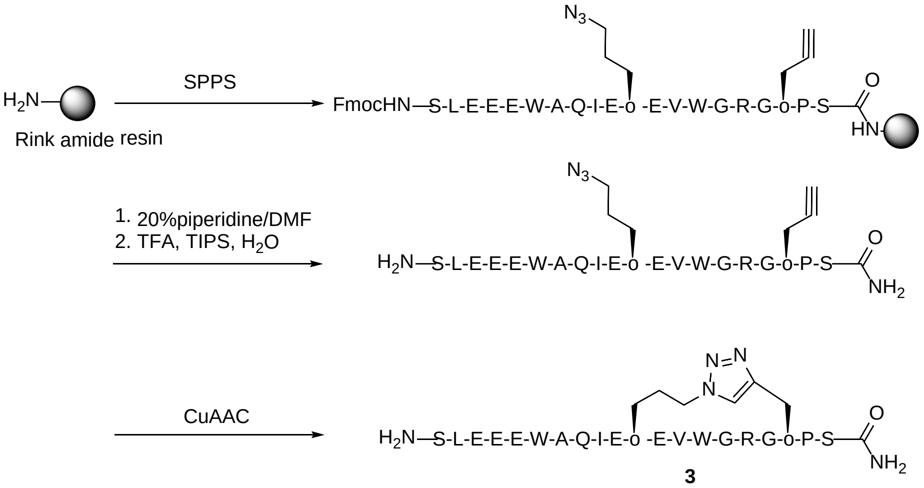

3

Scheme S3. Preparation peptide **3**. o denotes C^{α} atom of non-standard amino acids.

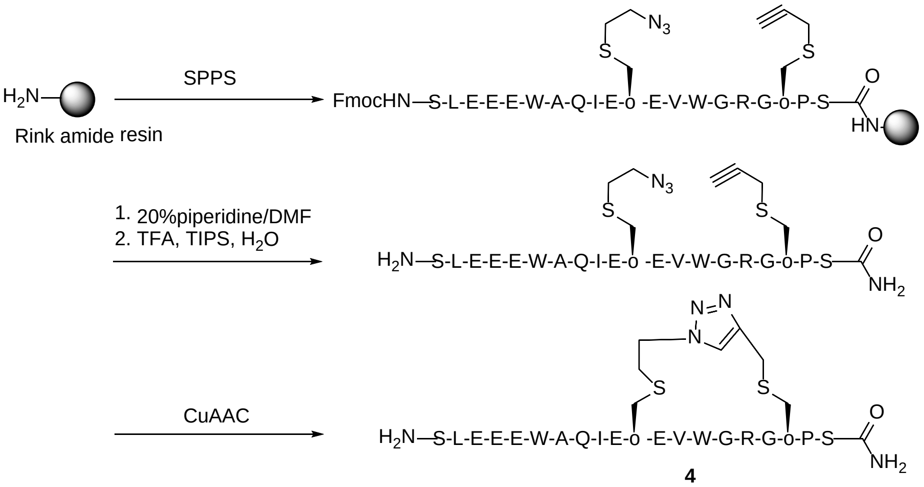

4

Scheme S4. Preparation peptide **4**. o denotes C^{α} atom of non-standard amino acids.

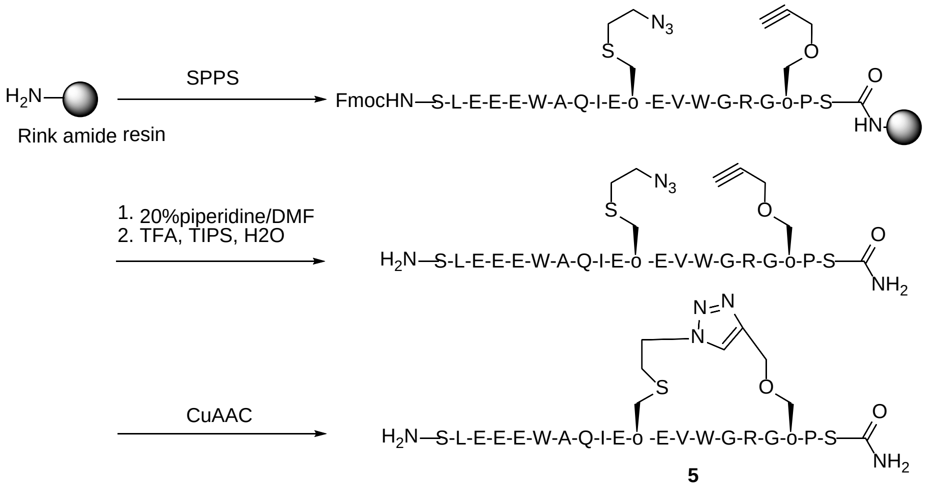

Scheme S5. Preparation peptide **5.** o denotes C^{α} atom of non-standard amino acids.

*Analytical chromatograms of peptides **1-5***

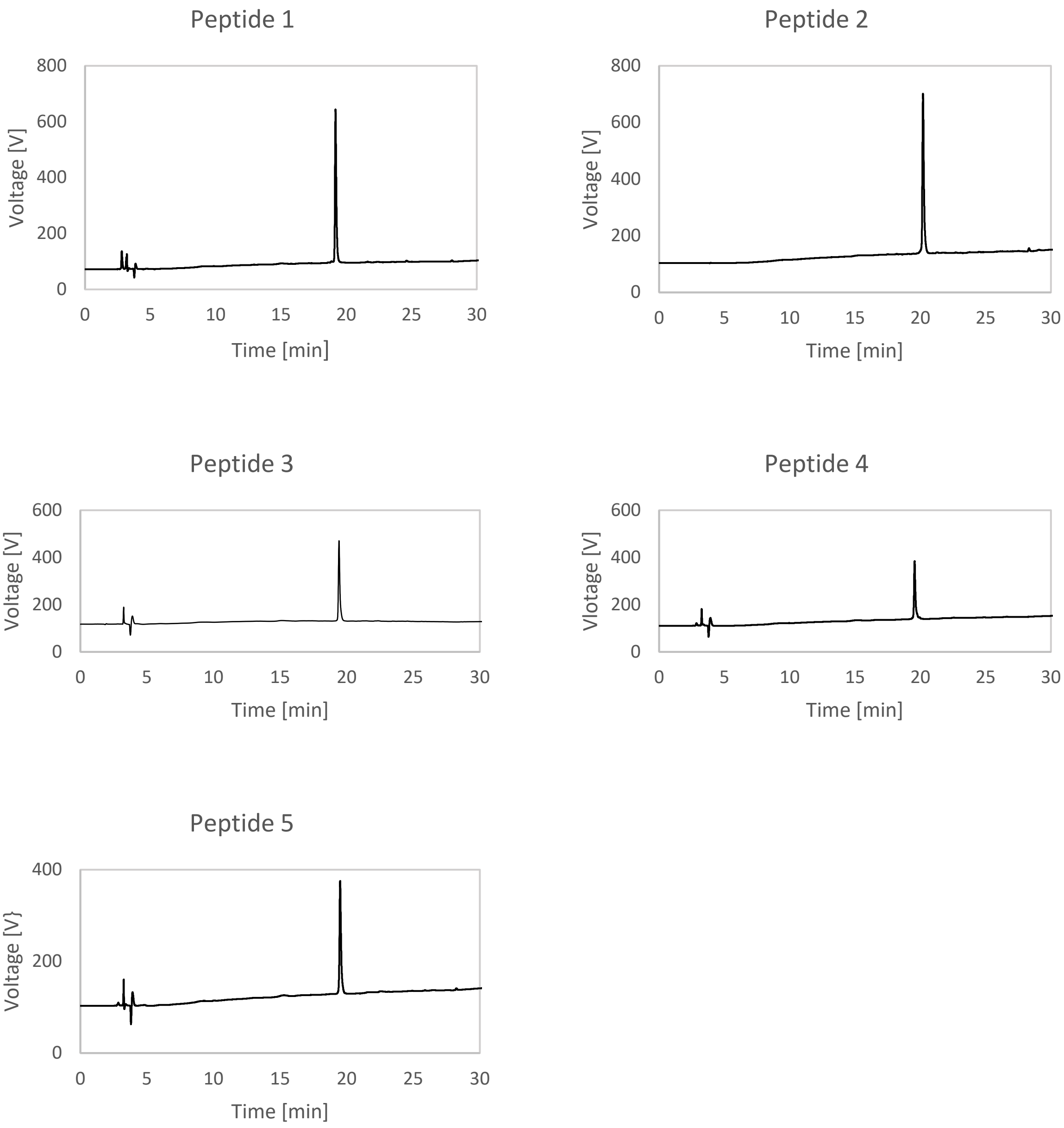

Figure **S10**. Analytical HPLC profiles of purified peptides **1-5**.

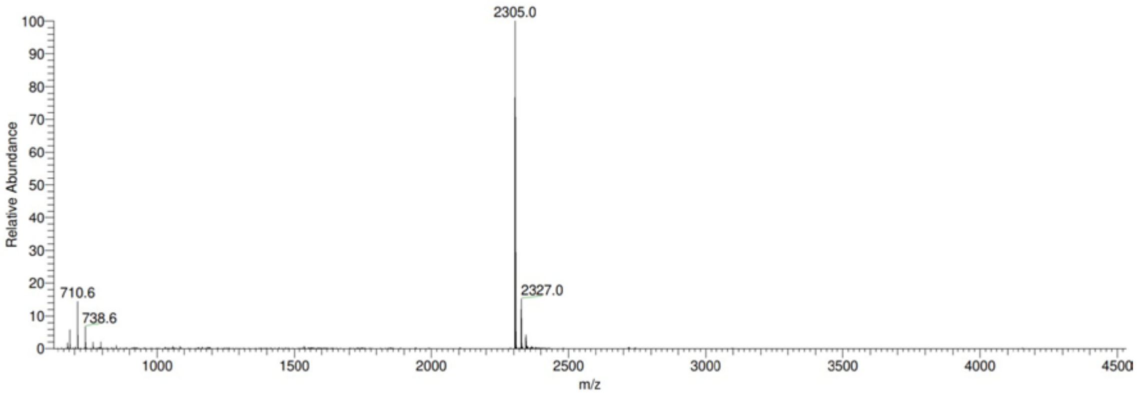

Figure S11. Mass spectrum of peptide **1**. Exact MH^+ expected 2305.0 ($C_{99}H_{145}N_{27}O_{33}S_2$).

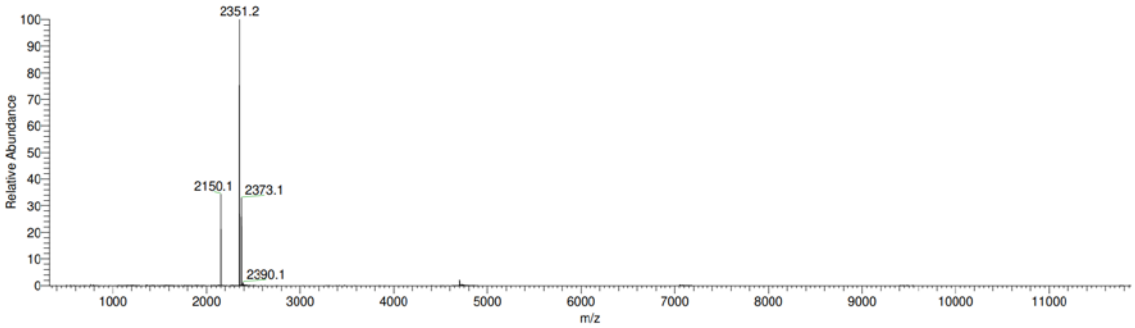

Figure S12. Mass spectrum of peptide **2**. Exact MH^+ expected 2351.2 ($C_{107}H_{159}N_{27}O_{33}$).

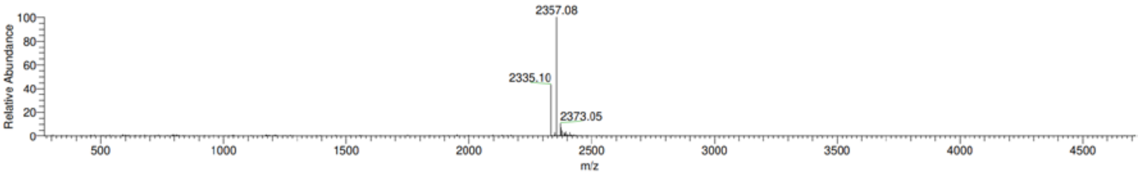

Figure S13. Mass spectrum of peptide **3**. Exact MH^+ expected 2336.1 ($C_{103}H_{150}N_{30}O_{33}$).

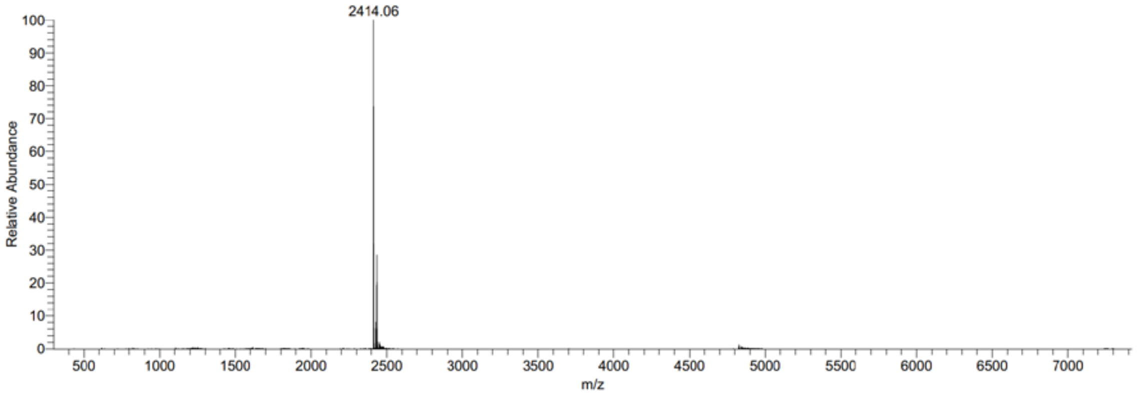

Figure S14. Mass spectrum of peptide **4**. Exact MH+ expected 2414.1 ($C_{104}H_{152}N_{30}O_{33}S_2$).

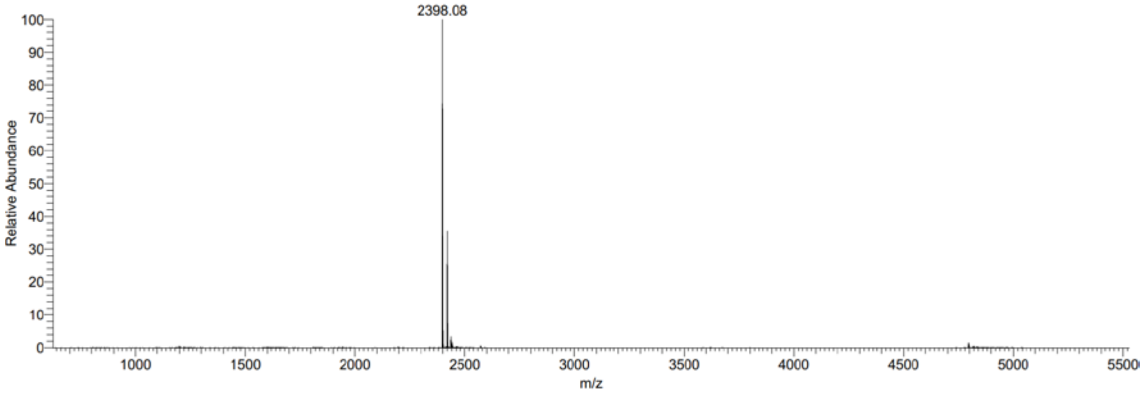

Figure S15. Mass spectrum of peptide **5**. Exact MH+ expected 2398.1 ($C_{104}H_{152}N_{30}O_{34}S$).

NMR data of peptides ***1-5***

Table S1. Proton NMR data of peptide **1** (600 MHz; in H_2O+D_2O 95:5+AcOD; pH = 3.0; T = 45 °C)

Residue	NH	*J*(NH,Hα)	Hα	Hβ	Hγ	Others
**Ser-1**	*a*	*a*	4.20	4.04	--	--
**Leu-2**	8.64	5.6	4.34	1.59	1.59	γ-Me: 0.90; 0.86
**Glu-3**	8.33	6.0	4.25	2.05; 1.98	2.44	--
**Glu-4**	8.05	6.3	4.25	1.98; 1.93	2.41	--
**Glu-5**	8.16	6.1	4.26	2.06	2.45; 2.36	--
**Trp-6**	8.10	3.8	4.53	3.33	--	H2: 7.24; H4: 7.56; H5: 7.12; H6: 7.18; H7: 7.42; N1H: 9.92
**Ala-7:**	7.98	4.2	4.05	1.37	--	--
**Gln-8**	8.23	3.8	4.08	1.93	2.25	$CONH_2$: 7.27; 6.70
**Ile-9**	7.80	5.8	3.84	1.85	1.54; 1.16	β-Me: 0.83; γ-Me: 0.81
**Glu-10**	8.24	4.5	4.14	2.05	2.52; 2.46	--
**Xxx-11**	7.96	5.8	4.54	3.07; 2.80	--	--
**Glu-12**	7.80	6.0	4.13	2.12	2.38	--
**Val-13**	8.19	7.8	3.76	1.58	0.76; 0.12	--
**Trp-14**	7.38	7.6	4.83	3.44; 3.28	--	H2: 7.18; H4: 7.64; H5: 7.06; H6: 7.20; H7: 7.44; N1H: 9.99
**Gly-15**	8.46	5.8	3.93; 3.78	--	--	--
**Arg-16**	7.75	8.3	4.53	1.80	1.61	Hδ: 3.13; NHε: 7.066
**Gly-17**	7.83	5.8	4.03	--	--	--
**Zzz-18**	7.80	6.0	4.48	3.06	--	--
**Pro-19**	--	--	4.45	2.28; 2.01	1.95	Hδ: 3.824; 3.679
**Ser-20-NH_2**	8.25	4.0	4.36	3.85	--	$CONH_2$: 7.06; 6.46

[a] Undetermined value

Table S2. Proton NMR data of peptide **2** (600 MHz; in H_2O+D_2O 95:5 + AcOD; pH = 3.0; T = 45 °C)

**Residue**	**NH**	**Hα**	**Hβ**	**Hγ**	**Others**
**Ser-1**	*a*	4.11	4.04	--	--
**Leu-2**	8.71	4.57	1.58	1.58	γ-Me: 0.88; 0.83
**Glu-3**	8.35	4.13	1.97	2.39	--
**Glu-4**	7.92	4.18	1.97	2.39	--
**Glu-5**	8.04	4.19	2.04	2.34	--
**Trp-6**	8.08	4.47	3.27	--	H2: 7.15; H4: 7.46; H5: 7.00; H6: 7.07; H7: 7.35; N1H: 9.80
**Ala-7:**	7.91	4.01	1.36	--	--
**Gln-8**	7.71	4.04	1.89	2.06	$CONH_2$: 7.22; 6.66
**Ile-9**	7.96	3.75	1.80	1.76	β-Me: 0.77; γ-Me: 0.38
**Glu-10**	8.04	4.19	2.05	2.42; 2.34	--
**Xxx-11**	7.999	--	1.12; 1.24; 1.42 (Me)	1.79	Hδ: 1.96; 1.88; Hε: 5.43 – 5.27
**Glu-12**	7.77	4.08	2.09	2.34	--
**Val-13**	7.68	3.80	1.80	0.74; 0.72	--
**Trp-14**	8.05	4.64	3.36; 3.14	--	H2: 7.15; H4: 7.60; H5: 7.02; H6: 7.10; H7: 7.37; N1H: 9.80
**Gly-15**	7.99	3.95	--	--	--
**Arg-16**	7.70	4.57	1.88; 1.72	1.59	Hδ: 3.13; NεH: 7.08
**Gly-17**	8.43	3.89; 3.83	--	--	--
**Zzz-18**	7.81	--	1.12; 1.24; 1.42 (Me)	1.79	Hδ: 1.96; 1.88; Hε: 5.43 – 5.27
**Pro-19**	--	4.43	2.24; 1.95	1.85	Hδ: 3.68; 3.48
**Ser-20-NH_2**	7.93	4.36	3.90	--	$CONH_2$: 7.34; 7.10

[a] Undetermined value.

Table S3. Proton NMR data of peptide **3** (600 MHz; in H_2O+D_2O 95:5 + AcOD; pH = 3.0; T =25 °C)

**Residue**	**NH**	**Hα**	**Hβ**	**Hγ**	**Others**
**Ser-1**	*a*	4.21	4.08; 4.04	--	--
**Leu-2**	8.79	4.31	1.59	1.59	γ-Me: 0.91; 0.86
**Glu-3**	8.47	4.20	2.03	2.44	--
**Glu-4**	*a*	*a*	*a*	*a*	--
**Glu-5**	*a*	*a*	*a*	*a*	--
**Trp-6**	8.13	4.23	3.31	--	H2: 7.21; H4: 7.57; H5: 7.06; H6: 7.18; H7: 7.42; N1H: 9.98
**Ala-7:**	8.04	4.04	1.38	--	--
**Gln-8**	8.22	4.02	1.85	2.14	$CONH_2$: 7.555; 7.144
**Ile-9**	7.89	3.85	1.54	1.14	β-Me: 0.79; γ-Me: 0.77
**Glu-10**	8.24	4.23	2.08	2.46; 2.38	--
**Xxx-11**	7.88	4.12	1.92; 1.87	1.79; 1.69	Hδ: 2.39; 2.19; =CH-(triazine): 7.78
**Glu-12**	8.12	4.14	2.05	2.52	--
**Val-13**	7.98	3.79	1.77	0.77; 0.41	--
**Trp-14**	7.75	4.74	3.37; 3.21	--	H2: 7.13; H4: 7.53; H5: 7.08; H6: 7.13; H7: 7.40; N1H: 9.98
**Gly-15**	7.84	3.95	--	--	--
**Arg-16**	7.93	4.32	1.60	1.82	Hδ: 3.13
**Gly-17**	8.38	3.83	--	--	--
**Zzz-18**	8.26	4.82	3.20; 3.00	--	--
**Pro-19**	--	4.47	2.30; 1.98	1.98	Hδ: 3.73; 3.59
**Ser-20-NH_2**	8.48	4.41	3.88	--	$CONH_2$: 7.43; 6.84

[a] Undetermined value

Table S4. Proton NMR data of peptide **4** (600 MHz; in H_2O+D_2O 95:5 + AcOD; pH = 3.0; T =25 °C)

**Residue**	**NH**	**Hα**	**Hβ**	**Hγ**	**Others**
**Ser-1**	*a*	4.18	4.02	--	--
**Leu-2**	8.71	4.34	1.59	1.59	γ-Me: 0.91; 0.87
**Glu-3**	8.42	4.24	2.06; 2.02	2.42	--
**Glu-4**	8.33	4.24	2.04	2.47; 2.42	--
**Glu-5**	8.19	4.22	1.87	2.37	--
**Trp-6**	7.99	4.65	3.32; 3.22	--	H2: 7.18; H4: 7.56; H5: 7.10; H6: 7.20; H7: 7.45; N1H: 10.06
**Ala-7:**	7.98	4.12	1.31	--	--
**Gln-8**	7.97	4.18	2.10; 2.04	2.34	$CONH_2$: *a*
**Ile-9**	7.92	4.01	1.86	1.48	β-Me: 0.86; γ-Me: 0.83
**Glu-10**	8.24	4.20	1.95	2.29	--
**Zzz-11**	8.12	4.45	2.96; 2.74	--	S-CH_2: 2.82; 2.73; CH_2-N: 2.97; N-CH=: 7.90; -CH_2-S: 4.51
**Glu-12**	8.26	4.23	2.00	2.38; 2.31	--
**Val-13**	7.86	3.95	1.87	0.82; 0.65	--
**Trp-14**	8.08	4.36	3.29; 2.81	--	H2: 7.24; H4: 7.56; H5: 7.10; H6: 7.20; H7: 7.45; N1H: 10.05
**Gly-15**	8.42	3.83	--	--	--
**Arg-16**	8.12	4.35	1.88; 1.72	1.63	Hδ: 3.18; NHε: 7.17
**Cys-17**	7.99	3.92; 3.86	--	--	--
**Zzz-18**	8.12	4.43	2.96; 2.73	--	S-CH_2: 2.82; 2.73; CH_2-N: 2.97; N-CH=: 7.90; -CH_2-S: 4.51
**Pro-19**	--	4.39	2.23	1.95	Hδ: 3.58; 3.51
**Ser-20-NH_2**	8.17	4.37	3.86; 3.81	--	$CONH_2$: 7.42; 6.80

[a] Undetermined value

Table S5. Proton NMR data of peptide **5** (600 MHz; in H_2O+D_2O 95:5 + AcOD; pH = 3.0; T =25 °C)

**Residue**	**NH**	**J(N,α)**	**Hα**	**Hβ**	**Hγ**	**Others**
**Ser-1**	*a*	*a*	4.18	4.01	--	--
**Leu-2**	8.70	6.5	4.35	1.58	1.583	γ-Me: 0.91; 0.87
**Glu-3**	8.41	6.3	4.24	2.03; 1.95	2.419	--
**Glu-4**	8.18	6.8	4.22	1.87	2.357	--
**Glu-5**	8.25	7.0	4.22	1.90	2.313	--
**Trp-6**	8.04	6.4	4.60	3.29; 3.21	--	H2: 7.23; H4: 7.56; H5: 7.09; H6: 7.19; H7: 7.45; N1H: 10.046
**Ala-7:**	7.97	5.6	4.18	1.30	--	--
**Gln-8**	7.98	7.1	4.19	2.08; 2.02	2.334	$CONH_2$: 7.45; 7.11
**Ile-9**	7.93	7.1	4.03	1.85	1.478	β-Me: 0.82; γ-Me: 0.86
**Glu-10**	8.24	6.2	4.22	1.98	2.367	--
**Xxx-11**	8.11	~7.1	4.33	2.80	--	--
**Glu-12**	8.30	6.7	4.24	2.02; 1.94	2.437; 2.397	--
**Val-13**	7.83	7.8	3.95	1.88	0.806; 0.680	--
**Trp-14**	8.04	6.4	4.60	3.30; 3.21	--	H2: 7.17; H4: 7.56; H5: 7.10; H6: 7.19; H7: 7.45; N1H: 10.05
**Gly-15**	8.40	6.1	3.90	--	--	--
**Arg-16**	8.10	7.5	4.31	1.87; 1.72	1.60	Hδ: 3.16
**Gly-17**	8.08	6.1	3.89; 3.83	--	--	--
**Zzz-18**	8.11	7.1	4.74	3.76; 3.71	--	--
**Pro-19**	--	--	4.40	2.26; 2.00	1.966	Hδ: 3.654; 3.564
**Ser-20-NH_2**	8.20	7.3	4.36	3.85; 3.80	--	$CONH_2$: 7.43; 6.81

[a] Undetermined value

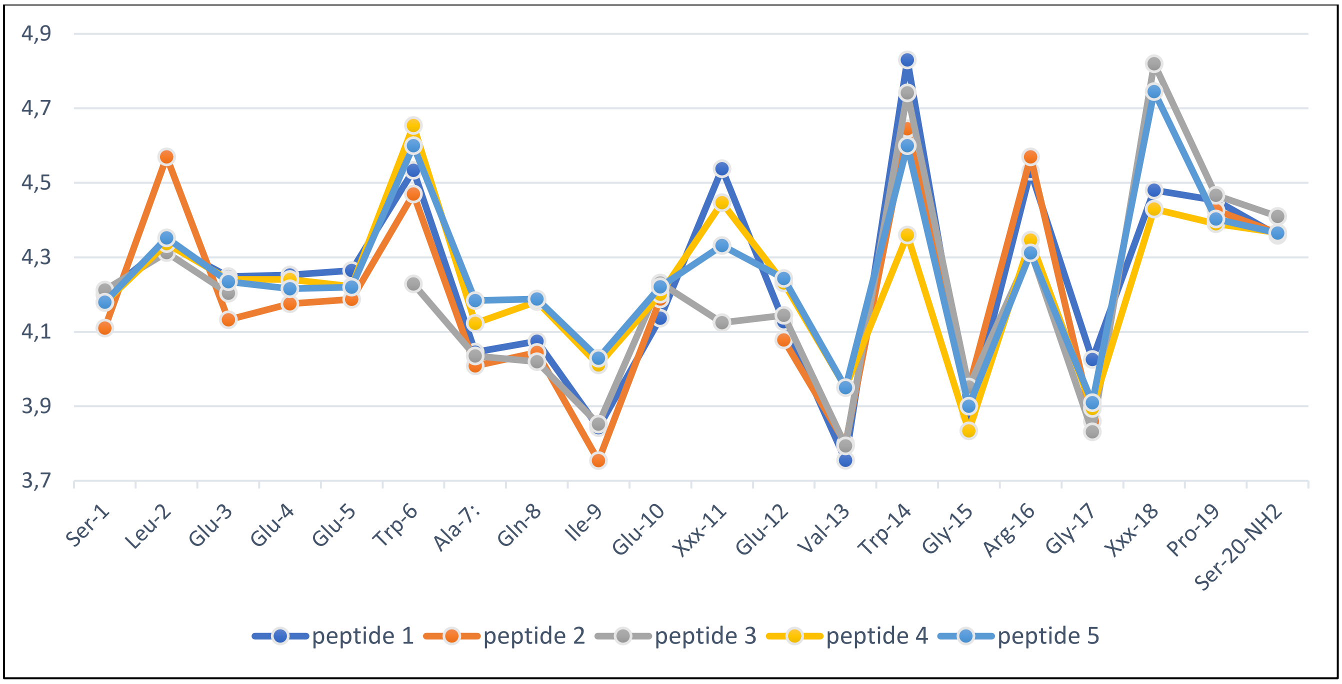

Figure S16. Comparison of Hα chemical shifts in peptides **1-5.**

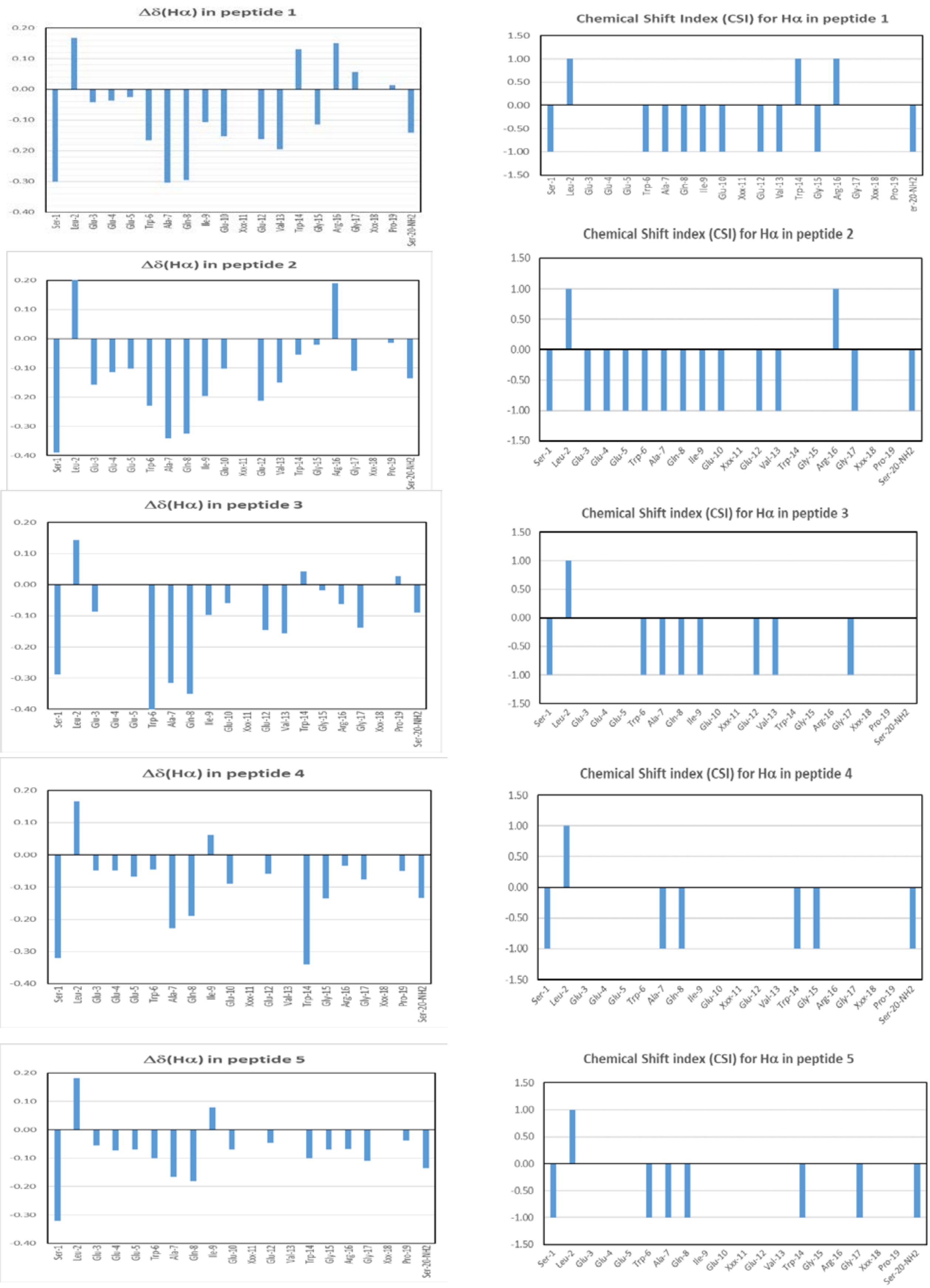

Figure S17. The Δδ(Hα) values (left) and corresponding CSI diagrams of peptides **1**- **5** (right).

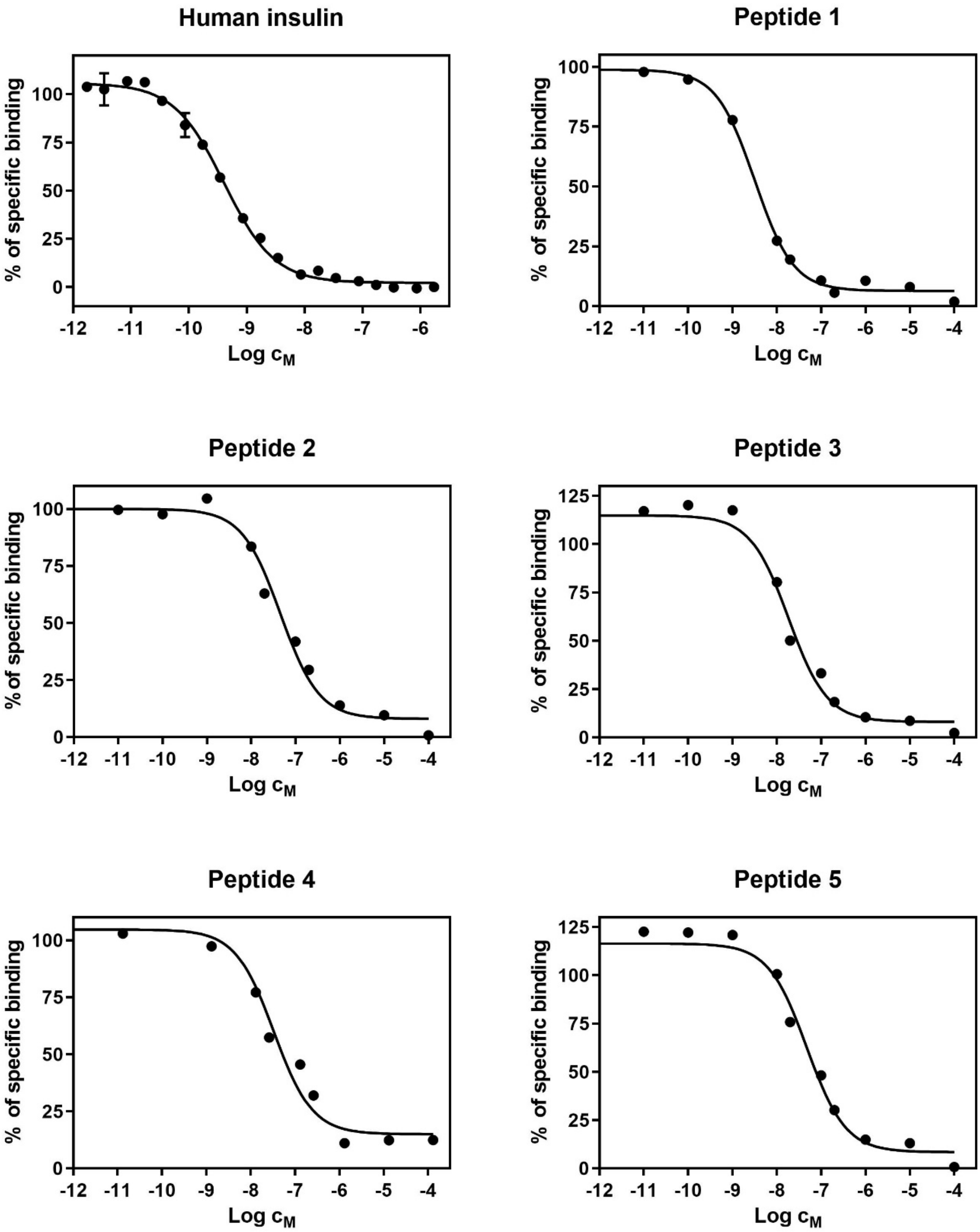

Figure S18. Representative binding curves of human insulin and peptides **1**-**5** on IR-A.

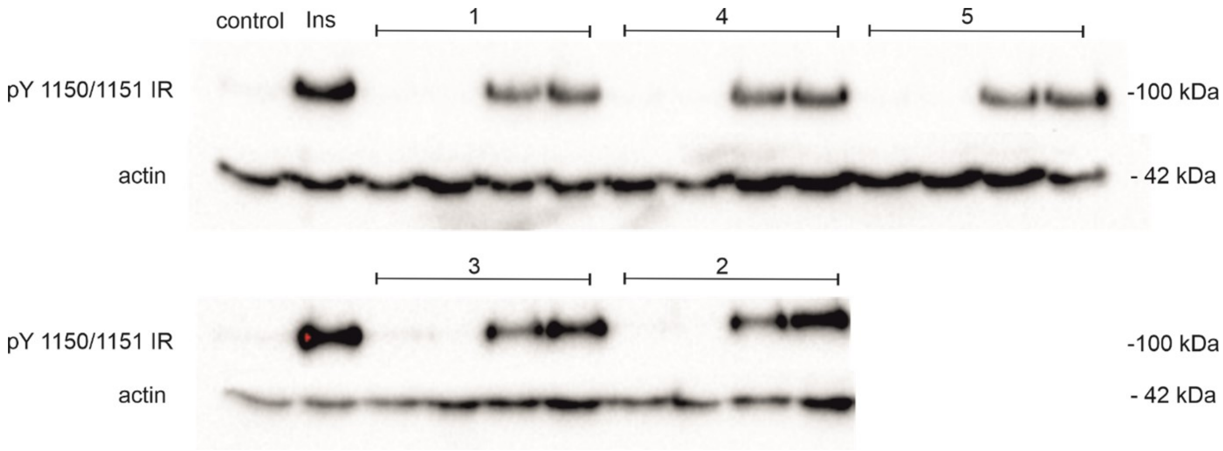

Figure S19. Representative Western blots for the abilities of peptides to stimulate IR-A phosphorylation and to antagonize insulin-stimulated IR-A phosphorylation. Cells were stimulated with 10 μM and 5 μM ligands alone, or in the presence of 10 nM insulin for 10 min. Control is no stimulation, Ins is 10 nM insulin. Each analog was tested in 4 wells as denoted by the line as 10 μM, 5 μM, 10 μM + Ins, 5 μM + Ins. Membranes were cut at 75 kDa and 50 kDa standards, and respective parts were developed with anti-phospho-IGF-1Rβ (Tyr1135/1136)/IRβ (Tyr1150/1151) antibody (Mr above 75 kDa) and with anti-actin antibody (Mr below 50 kDa).

Supplementary references

1 Sminia TJ and Pedersen DS. Azide- and alkyne-functionalised alpha- and beta(3)-amino acids. *Synlett* 2012: 2643-2646.

2 Hiremathad A, Chand K, Esteves AR, Cardoso SM, Ramsay RR, Chaves S, Keri RS and Santos MA. Tacrine-allyl/propargylcysteine-benzothiazole trihybrids as potential anti-Alzheimer's drug candidates. *RSC Advances* 2016, **6**: 53519-53532.

3 Fumagalli G, Carbajo RJ, Nissink JWM, Tart J, Dou RX, Thomas AP and Spring DR. Targeting a Novel KRAS Binding Site: Application of one-component stapling of small (5-6-mer) peptides. *J Med Chem* 2021, **64**: 17287-17303.

4 Gongora-Benitez M, Mendive-Tapia L, Ramos-Tomillero I, Breman AC, Tulla-Puche J and Albericio F. Acid-labile Cys-protecting groups for the Fmoc/tBu strategy: filling the gap. *Org Lett.* 2012, **14**: 5472-5475.

5 Picha J, Budesinsky M, Machackova K, Collinsova M and Jiracek J. Optimized syntheses of Fmoc azido amino acids for the preparation of azidopeptides. *J Pept Sci* 2017, **23**: 202-214.

6 Lubos M, Mrazkova L, Gwozdiakova P, Picha J, Budesinsky M, Jiracek J, Kaminsky J and Zakova L. Functional stapled fragments of human preptin of minimised length. *Org Biomol Chem.* 2022, **20**: 2446-2454.
